# eLemur: A cellular-resolution 3D atlas of the mouse lemur brain

**DOI:** 10.1073/pnas.2413687121

**Published:** 2024-12-04

**Authors:** Hyungju Jeon, Jiwon Kim, Jayoung Kim, Yoon Kyoung Choi, Chun Lum Andy Ho, Fabien Pifferi, Daniel Huber, Linqing Feng, Jinhyun Kim

**Affiliations:** ^a^Brain Science Institute, Korea Institute of Science and Technology, Seoul 02792, South Korea; ^b^Division of Bio-Medical Science & Technology, Korea Institute of Science and Technology-School, University of Science and Technology, Seoul 02792, South Korea; ^c^Department of Computer Science and Engineering, Korea University, Seoul 02841, South Korea; ^d^Department of Basic Neurosciences, University of Geneva, Geneva 1205, Switzerland; ^e^Musée National d’Histoire Naturelle, Adaptive Mechanisms and Evolution, UMR7179—CNRS, Paris 75005, France; ^f^Korea Institute of Science and Technology-Sungkyunkwan University Brain Research Center, Sungkyunkwan University Institute for Convergence, Sungkyunkwan University, Suwon 16419, South Korea

**Keywords:** Gray mouse lemur (*Microcebus murinus*), 3D brain atlas, cellular resolution, website resources

## Abstract

The gray mouse lemur (*Microcebus murinus*), one of the smallest living primates, emerges as a promising model organism for neuroscience research. This is due to its genetic similarity to humans, its evolutionary position between rodents and humans, and its primate-like features encapsulated within a rodent-sized brain. Despite its potential, the absence of a comprehensive reference brain atlas impedes the progress of research endeavors in this species, particularly at the microscopic level. Existing references have largely been confined to the macroscopic scale, lacking detailed anatomical information. Here, we present eLemur, a unique resource, comprising a repository of high-resolution brain-wide images immunostained with multiple cell type and structural markers, elucidating the cyto- and chemoarchitecture of the mouse lemur brain. Additionally, it encompasses a segmented two-dimensional reference and 3D anatomical brain atlas delineated into cortical, subcortical, and other vital regions. Furthermore, eLemur includes a comprehensive 3D cell atlas, providing densities and spatial distributions of non-neuronal and neuronal cells across the mouse lemur brain. Accessible via a web-based viewer (https://eeum-brain.com/#/lemurdatasets), the eLemur resource streamlines data sharing and integration, fostering the exploration of different hypotheses and experimental designs using the mouse lemur as a model organism. Moreover, in conjunction with the growing 3D datasets for rodents, nonhuman primates, and humans, our eLemur 3D digital framework enhances the potential for comparative analysis and translation research, facilitating the integration of extensive rodent study data into human studies.

## Significance

The gray mouse lemur (*Microcebus murinus*) represents a promising model for neuroscience research, offering insights into brain structure and function due to its genetic and evolutionary proximity to humans and rodents. Our development of eLemur, a comprehensive three-dimensional (3D) digital brain atlas, fills critical gaps in microscopic data availability, enabling nuanced investigations at cellular resolutions. This resource not only enhances our understanding of the mouse lemur brain but also catalyzes broader neuroscience endeavors. By facilitating data sharing and integration, eLemur empowers researchers to explore different hypotheses and experimental designs. Moreover, its compatibility with existing neuroanatomy frameworks and the growing repository of 3D datasets positions eLemur as a pivotal tool for advancing basic and translational neuroscience studies.

Model organisms have been pivotal in propelling advancements in biology, especially in the realm of modern neuroscience research (1–8). The laboratory mouse, in particular, has led to significant breakthroughs in genetics and cutting-edge technologies (from gene editing to automated behavioral analyses), shaping our understanding of the brain (9–14). However, despite these strides, challenges persist in translating rodent-based findings to primate biology, behavior, and human-relevant diseases. Neuroscience research often is conducted under the assumption that findings generalize across species, but many brain areas, including the neocortex, have species-specific functional organizations. Recognizing the need for diverse animal models and a comparative approach, there is growing interest in expanding the portfolio of model species for next-generation neuroscience research (5, 7, 8, 15–18).

Among emerging candidates, the mouse lemur (*Microcebus murinus*) has drawn attention as a promising nonhuman primate model system (17, 19–24). Mouse lemurs are suggested as a practical and cost-efficient model for neuroscience research due to their unique attributes, such as its small rodent-like body and brain size, potential for rapid colony growth, and genetic proximity to humans. Moreover, the mouse lemur exhibits remarkable adaptability to laboratory settings and holds the potential for leveraging transferable tools developed for mice, positioning it as an advantageous primate model system that bridges the gap between primate and rodent studies (17). Studies employing this species increase, exploring comparable information and transferable experimental paradigms between the mouse lemur, primates, and rodents (22, 25–28). These endeavors support the potential of the mouse lemur for elucidating fundamental aspects of neurobiology, ultimately facilitating the translation of extensive rodent studies into organizational principles governing further complex nervous systems.

To establish the mouse lemur as a model organism, our understanding of its genetic and anatomical features is fundamental. Currently, ongoing genome sequencing projects aim to extensively map the mouse lemur genome (29), while single-cell resolution transcriptome projects strive to provide comprehensive molecular cell profiles (21). Additionally, classic Nissl stain-based stereotaxic atlases and MRI-based brain atlases have already been established (26, 30, 31). Nevertheless, these atlases, while informative, provide only a coarse view of anatomical structures lacking the spatial molecular and cellular details crucial for functional inference. Identifying brain subregions and specific cell types is essential for elucidating functional diversity, mapping neural circuits, and uncovering the cellular basis of neurological disorders. Ultimately, this knowledge will deepen our comprehension of brain function and dysfunction, guiding targeted therapeutic interventions (32).

In addressing this gap, we present a digital 2D/3D atlas of the mouse lemur brain with cellular resolution, incorporating advanced fluorescence immuno-labeling and computational imaging analysis. This digital atlas, named eLemur, is publicly accessible through our web-based viewer (https://eeum-brain.com/#/lemurdatasets). eLemur comprises 1) a repository of high-resolution brain-wide immuno-fluorescence images with multiple cell type and structural markers, revealing the cyto- and chemoarchitecture of the mouse lemur brain; 2) a 2D/3D brain atlas serving as a template, segmented into 54 brain subregions based on a hierarchical ontology; and 3) a 3D cell atlas providing region-by-region densities and positions of non-, neuronal, and parvalbumin (PV) expressing cells. Our cellular-resolution 3D atlas of the mouse lemur brain, eLemur, offers a resource for the neuroscience community and will facilitate future research bridging gaps in our understanding of rodents, nonhuman primates, and humans.

## Results

### Mouse Lemur Brain Atlas Pipeline and Data Resources.

**Fig. 1.**
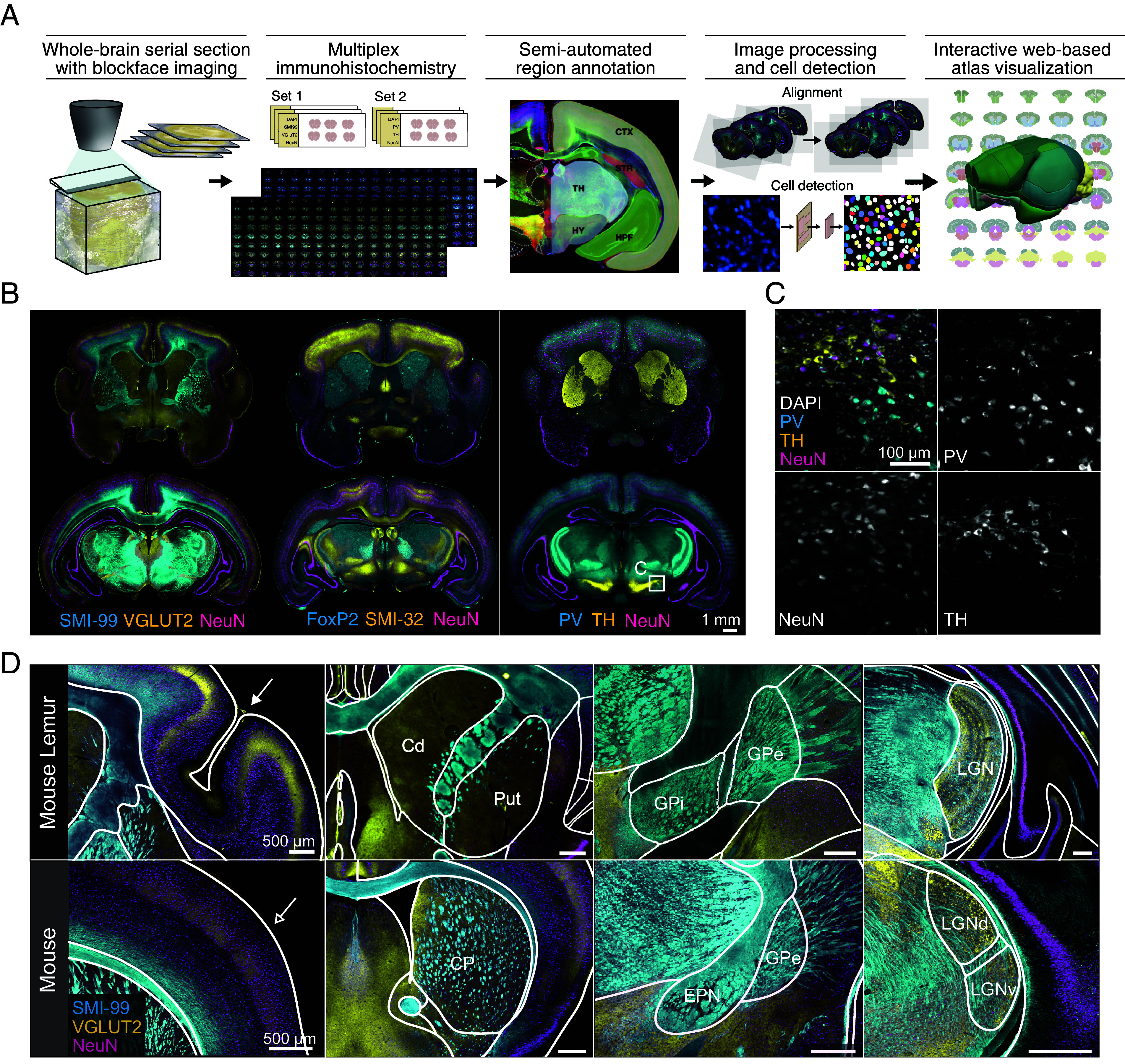
Atlas pipeline and anatomical features of the mouse lemur brain. (*A*) Illustration of the pipeline generating multiplex brain-wide immunofluorescence image datasets and constructing a 2D/3D atlas of the mouse lemur brain. (*B*) Representative immunofluorescence images showcasing multiplex cell type and structural markers, illustrating the cytoarchitecture of the mouse lemur brain. (*C*) High magnification images of the boxed area in *B*, displaying NeuN-, PV-, and tyrosine hydroxylase (TH)-positive cell types in the substantia nigra. (*D*) Comparison of anatomical features between the mouse lemur and mouse brains. The mouse lemur brain exhibits anatomical homologies shared with other primates, including cortical folding (*Top Left*, indicated by an arrowhead), the separated caudate and putamen, as well as the GPi (*Top Middle*), and the laminar structure of the LGN (*Top Right*). These features are absent in the mouse brain (*Bottom*).

To construct a high-resolution 3D atlas of the mouse lemur brain, we devised a pipeline comprising several key steps: whole-brain sectioning with block face imaging, brain-wide multiplex immunolabeling, and fluorescence imaging with subsequent imaging processing, brain region annotation, 2D image alignment, cell detection analysis leading to 3D atlas generation, and implementation of an interactive web-based visualization platform (Fig. 1*A* and *Materials and Methods*).

**Fig. 2.**
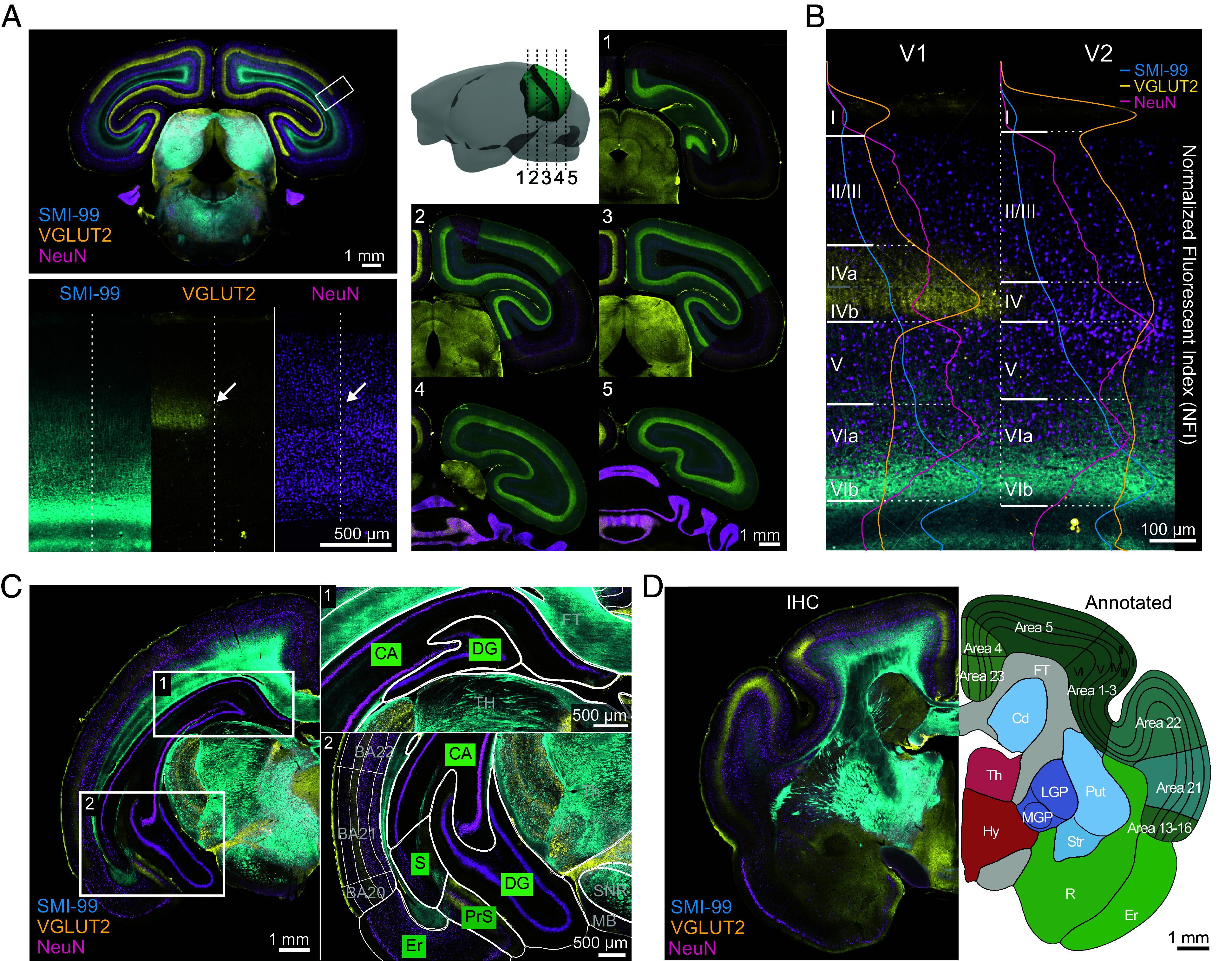
Delineating brain structures and annotating a 2D reference atlas. (*A*) Representative coronal immunofluorescence image with three marker staining: SMI-99, VGLUT2, and NeuN. Enlarged images of the boxed area at the V1-V2 border (*Left*). The boundary between V1-V2, indicated by a dashed line, is defined by the sharp transition of VGLUT2 and NeuN signals (indicated by an arrow). The progression of the V1 area across the longitudinal axis (anterior–posterior) is shown with manual annotations in green (*Right*). (*B*) Cortical layers in V1 and V2 are defined by curated intensity and density of SMI-99, VGLUT2, and NeuN signals. The intensity plot of normalized fluorescence index along a perpendicular cortical column in V1 and V2 for each marker—SMI-99 (cyan), VGLUT2 (yellow), and NeuN (magenta)—is overlaid to demonstrate the laminar structure. (*C*) Hippocampal substructures, including dentate gyrus, subiculum, and entorhinal cortex, are identified and segmented using multiplexed immunosignals. (*D*) Representative 2D coronal section showing full annotations (*Right*) alongside the original immunofluorescence image (*Left*).

First, we explored suitable markers capable of delineating brain structures, axonal projections, and cell types compatible with mouse lemur brain tissue. Following a thorough comparison with mouse brain tissue, we selected a total of seven markers, along with DAPI, exhibiting high specificity in the mouse lemur brain to generate brain-wide fluorescence immunohistochemistry (IHC) data (SI Appendix, Table S1). The mouse lemur brains were coronally sectioned in 50 µm thickness, obtaining approximately 360 sections per brain. We conducted multiplex immunofluorescence staining with combinations of markers on serial coronal sections in order to intricately delineate the structural features of brain subregions (*Materials and Methods* and SI Appendix, Fig. S1). For subsequent registration of immunofluorescence images in the step of 3D atlas generation, the surface of the brain tissue block was captured using a digital camera before each sectioning process (referred to as block face image) (33). The high-resolution combinatorial multiplex immunofluorescence datasets, with a resolution of 0.65 µm in x-y dimensions, enabled the exploration of the intricate cyto- and chemoarchitecture of the mouse lemur brain. Immunofluorescence signals from the neuron-specific nuclear protein (NeuN), coupled with DAPI counterstaining, provided an overview of each the neuronal and non-neuronal population across different brain regions. Moreover, datasets obtained from PV, TH, and Forkhead box protein P2 staining revealed the spatial distribution of diverse cell types across the brain, thereby facilitating the analysis of distinct neural components (Fig. 1 *B* and *C*). Furthermore, TH signals were instrumental in characterizing the basal ganglia (BG), a group of subcortical nuclei implicated in motor control and movement disorders. Additionally, signals derived from vesicular glutamate transporter 2 (VGLUT2) and myelin basic protein (SMI-99) allowed for the differentiation of subregional structures in the cortex, hippocampus, and sensory thalamus (Figs. 1*B* and 2). These selected markers collectively serve as neuroscientific probes, enabling the investigation of distinct neural structures of the mouse lemur brain.

Through our initial investigation, we uncovered anatomical homologies shared across primate species, underscoring the mouse lemur’s potential as a bridging model between rodent and primate neuroscience studies (Fig. 1*D* and SI Appendix, Fig. S2).

Of notable significance is cortical folding, the presence of a layer IV (strongly suggesting the presence of a granular frontal cortex) (34), and the caudate nucleus and putamen separated by a distinct fiber tract—all features commonly observed in primate brains (35). While in rodents, these structures often appear fused into a unified striatum, in primates, they serve distinct roles in goal-directed behavior and sensorimotor coordination (36). Additionally, we observed the globus pallidus interna (GPi) as a distinct structure adjacent to the globus pallidus externa (GPe), a configuration differing from rodents where the entopeduncular nucleus is considered a debated homolog of the primate GPi (37, 38). The lateral geniculate nucleus (LGN), also known as the visual thalamus in mouse lemurs, exhibits a distinct six-layered structure, indicating a sophisticated visual information processing system that is common among primates but absent in rodents. Furthermore, in mouse lemurs, as in primates, the primary visual cortex exhibits a distinct division of layer IV into IVa and IVb (Fig. 2 *A* and *B*).

Each of these sublayers receives primary inputs from the LGN, conveying information from the contralateral and ipsilateral eye. This characteristic is unique to primates and is also observed in mouse lemurs, in contrast to rodents, where a single layer IV receives thalamic inputs (25, 39). Our primary objective was to establish a repository for brain-wide immunofluorescence datasets, aiming to offer fundamental insights into the intricate neural architecture and molecular characteristics of the mouse lemur brain. These findings underscore the mouse lemur’s potential as a promising model organism for understanding neural complexities across primate species. In conjunction with the previous study revealing hundreds of primate-specific gene expressions absent in mice (21), these brain anatomical findings highlight the mouse lemur’s unique position as an intermediary model between rodents and primates, offering broader translational implications for understanding human brain functions and associated disorders.

### Charting Brain Structures: Annotating a 2D Reference Atlas.

To generate a 2D Reference Atlas of the mouse lemur brain, we annotated a single reference specimen by drawing on its stained images, as traditionally done for the mouse and human (40, 41). The single reference brain was coronally sectioned and alternately divided into two staining sets. Set 1 was stained with NeuN, SMI-99, VGLUT2, and DAPI, while Set 2 was stained with NeuN, PV, TH, and DAPI. The 2D-immunofluorescence images were aligned to corresponding parts in the block face image via 2D rigid multiscale registration, followed by shading artifact correction (SI Appendix, Fig. S3). Brain structures were segmented based on their cellular architectures through these multiple markers (Fig. 2 and Movies S1 and S2), employing semiautomated structure annotation with a priority on preserving data in its original image state with minimal computational processing. This approach significantly reduced manual processing time, comprising steps of sparse manual annotations of the 13 major brain structures (neocortex, hippocampal formation, remaining unspecified cortical regions, striatum, pallidum, thalamus, hypothalamus, midbrain, pons, medulla, cerebellum, fiber tracts, and ventricular system) on the right hemisphere of alternate coronal images, training a deep learning-based region segmentation using the manual annotations for automatic annotation of the entire dataset, and detailed manual annotation for refinement and finer subregions (SI Appendix, Fig. S4). Manual annotations were facilitated by custom software featuring a graphical user interface (GUI) for easy annotation using cubic spline curves defined by control points, which allowed for detailed refinement. For further subregion delineations, we incorporated additional reference information, including a different set of multiplex immunofluorescence signals with brightfield images, the Nissl-based Bons atlas (30), and an MRI-based atlas (26) (Fig. 2*B* and SI Appendix, Fig. S5). This integration of diverse datasets improved fine region boundary identification and enhanced structural delineation (Fig. 2*D*). Subregion annotations focused on cortical layers, the hippocampus, and the BG, considering mouse lemurs’ specific attributes and their important functions.

#### Cortical areas and layers.

Given their important functions, we aimed for a finer level of annotations for cortical areas. The delineation of cortical layers is crucial for gaining insights into the functional specialization of different layers, which contributes to our understanding of neural circuits and their role in complex cognitive processes. Additionally, precise delineation facilitates comparative studies across species, shedding light on evolutionary adaptations and differences in brain function and behavior. The neocortex’s laminar structure, characterized by distinct molecular compositions and connectivity (42–45), poses challenges in delineating cortical layers based solely on cellular features, such as those indicated by DAPI and NeuN staining. To address this challenge, we combined cellular features from DAPI and NeuN with layer-specific synaptic terminal distribution indicated by VGLUT2, and integrated annotations from the Bons atlas (30) to accurately annotate cortical layers (Fig. 2 *A* and *B*). Utilizing VGLUT2 signals was useful in identifying sublayers, such as the dense concentrations in layer IV and blobs of layer 3 in the primary visual cortex (V1 or Brodmann area 17) and secondary visual cortex (V2 or Brodmann area 18) (25, 46, 47). Additionally, VGLUT2 signals helped distinguish sublayers IVa and IVb, with a narrow layer of high cell density and dense VGLUT2-labeled axon terminals in layer IVb and less so in IVa (Fig. 2*B*). Furthermore, we incorporated the MRI-based atlas (26) to delineate the Brodmann areas, which are specific regions of the cerebral cortex in humans and other primates. This process involved initializing the population averaged T2-weighted MRI template and corresponding labels to the block face volume through a series of 3D affine and nonlinear transformations, followed by slice-by-slice 2D deformable registration onto immunofluorescent images (SI Appendix, Fig. S5). The transferred annotations were then manually refined to ensure that the boundaries between regions aligned parallel to the radial direction. We reason that using Brodmann nomenclature facilitates comparative analysis with previously reported studies in both nonhuman primate and human research, where it remains widely used. Given the increasing focus on well-defined digital brain segmentations in the mouse brain, such as those provided by the Allen mouse brain ontology, we have included a table (SI Appendix, Table S3) that maps Brodmann areas to their corresponding functional names, serving as a thorough reference for cross-comparison. Our detailed annotation of cortical layers and delineation of Brodmann areas in the mouse lemur brain enhances our understanding of the intricate organization and functional specialization of the cerebral cortex across species.

#### Hippocampal formation.

As learning and memory are fundamental components of higher cognitive functions, the hippocampus (HP) has been extensively studied, with each subregion and layer displaying distinctive organization and function (48). We undertook segmentation of the hippocampal layers to lay anatomical groundwork for future investigations into hippocampal functions of mouse lemurs, which offer a more complex cognitive model (Fig. 2*C*). Similar to cortical annotations, we curated additional reference information and adopted the Bons atlas. We divided the hippocampal formation into five subregions: the Ammon’s horn (CA), subiculum (S), pre/para-subiculum (PrS), dentate gyrus (DG), and entorhinal cortex (Ent). Each hippocampal subregion consists of a distinguished laminar structure, which formed the basis of our segmentation. The dentate gyrus and Ammon’s horn were identified by densely packed layers of cell bodies, discernible from DAPI and NeuN signals. The boundary between the two regions was delineated by VGLUT2 and SMI-99 signals, along with blood vessels. The subiculum, located adjacent to the Ammon’s horn, was characterized by a relatively sparse pyramidal cell layer, distinctively observable through NeuN staining. The pre/parasubiculum and the entorhinal cortex were distinguished by discontinuation in layer structures.

#### BG.

Despite the well-preserved basic anatomy and connectivity of the BG across species, interspecific variations exist, notably evidenced from rodents to primates (37, 49). Our interspecies comparison, as depicted in Fig. 1*D* and SI Appendix, Fig. S2, reveals that the mouse lemur exhibits nuanced divergence in key component structures within the BG, reminiscent of those found in humans. To further elucidate these structures across the brain, we conducted comprehensive segmentation of the BG, targeting seven key regions implicated in movement control: the caudate, putamen, GPe, GPi, subthalamic nucleus (STh), substantia nigra pars reticulata (SNR), and pars compacta (SNC) (Fig. 1*D*). Employing both the multiplex stainings of VGLUT2/SMI-99/NeuN and TH/PV/NeuN sets, we utilized information primarily from PV and TH staining signals to delineate these substructures. The caudate nucleus and putamen, positioned at the anterior striatum, were separated by the internal capsule, with thin bridges of cells connecting the putamen to the caudate head. The GPi and GPe were located at the medioventral part of the medial- and lateral medullary lamina, respectively. The STh, positioned between the internal capsule and cerebral peduncle, was characterized by its distinctive, bright, lens-shaped nucleus across NeuN, PV, and SMI-32 staining. The pars compacta, located posterior to the STh, could be clearly distinguished from its neighboring regions through TH staining. Meanwhile, the pars reticulata, situated between the pars compacta and large fiber bundles (e.g., cerebral peduncle at the anterior and medial longitudinal fasciculus at the posterior), could be readily identified.

#### Ventricular structures and fiber tracts.

Utilizing immunofluorescence signals from SMI-99 and NeuN, complemented by brightfield contrast images and annotations from the Bons atlas, we delineated major fiber tracts including white matter structures including the corpus callosum, internal capsule, and cerebral peduncle (SI Appendix, Fig. S4A). These structures, distinguished by darker contrast in the brightfield channel and sparse NeuN signals, were further refined to include fine fiber tracts, such as the mammillothalamic tract within the thalamus. Additionally, within the ventricular system, the lateral, third, and fourth ventricles were annotated based on their distinguishable appearance as gray or empty areas, with ependymal cell-lined walls clearly visualized via DAPI staining, serving as a primary guide for delineation verification and modification (SI Appendix, Fig. S4B).

#### Hierarchical ontology and annotation.

Following thorough annotation of the mouse lemur brain, our next step was to optimize the ontology of its brain regions. Our goal was to enhance legibility and ensure compatibility with existing digital frameworks for other species, such as the Allen Reference Atlas (ARA) (50). We established consistent nomenclature by translating 239 structures from Latin names used in the Bons atlas to English and reconciling inconsistent nomenclatures and abbreviations (SI Appendix, Table S2). Our primary references included NeuroNames (51), a comprehensive neuroanatomical database, and the ARA of the mouse brain when standard names or acronyms were not available in NeuroNames.

To systematically define the relationships between brain regions, we organized a hierarchical ontology based on Brain maps 4.0 (52) and the Allen mouse brain ontology. We structured the mouse lemur brain ontology into a hierarchical tree with four levels. At the root level, divisions were made into gray matter (including cell groups and regions), white matter (fiber tracts), and the ventricular system. The gray matter was further categorized into the cerebrum, brainstem, and cerebellum at level 1, which were then subdivided into three hierarchical layers across levels 2 to 4 (SI Appendix, Table S4). We defined 13 major brain structures for annotation and further detailed subdivisions of key regions such as the cortical areas, hippocampal formation, and BG at level 4. To ensure intuitive comparison and compatibility with the widely used ARA, we adopted the color coding of the Allen Institute’s colormap throughout our analysis (53). A complete hierarchical tree of 54 regions annotated in the reference atlas with unique colors can be found in SI Appendix, Table S4.

### 3D Reference Atlas of the Mouse Lemur.

The demand for digital 3D atlases is increasing due to their significant advantages over traditional 2D slice-based atlases. These 3D models serve as comprehensive anatomical frameworks, facilitating the integration of findings from various modalities. Deriving precise 3D structures solely from a sequence of 2D atlas images is highly challenging despite provided stereotaxic coordinates and is prone to misalignment of experimental data due to variations in brain cutting angles. Thus, developing a precise 3D atlas is crucial for understanding the complex anatomy and organization of the mouse lemur brain structures. The eLemur 3D digital atlas addresses these challenges by providing a common reference model that integrates diverse experimental data and enables comparative analysis with 3D atlases of other species.

**Fig. 3.**
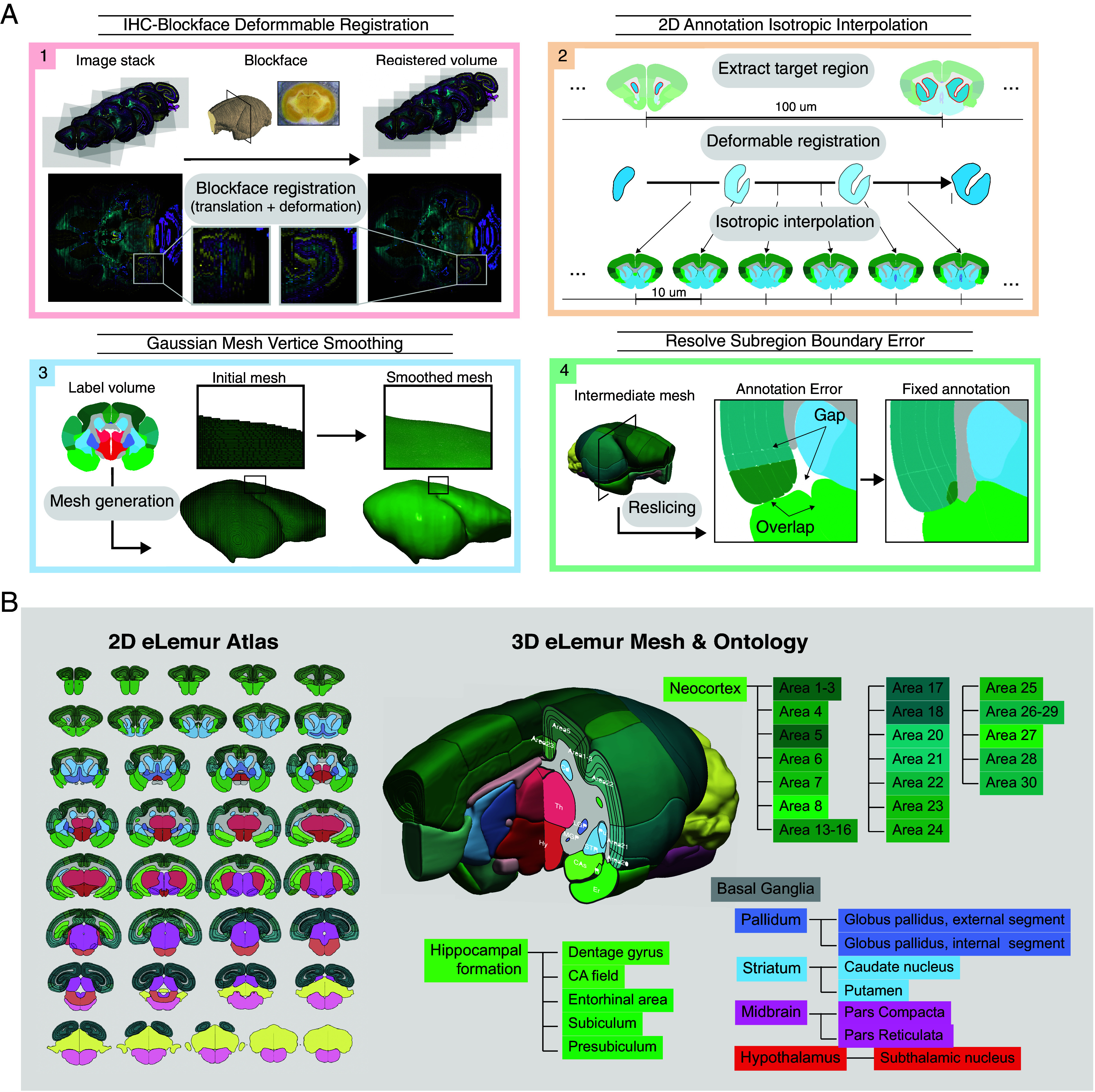
2D/3D reference atlas of the mouse lemur brain. (*A*) Diagram of the process for transforming from 2D to 3D, including block face registration, isotropic interpolation of 2D annotations, and generation of a 3D mesh of brain regions. (*B*) Representative serial annotated 2D atlas (*Left*) and the 3D eLemur mesh with the hierarchical ontology of annotated subregion structures (*Right*). Regions are color-coded based on the color scheme of corresponding structures from the Allen Mouse Brain Atlas.

Our 2D to 3D translation process began by registering histological images to corresponding block face images to correct tissue distortions and damages incurred during the sectioning and staining process (Fig. 3 and SI Appendix, Fig. S3).

Tissue areas from the block face images were semiautomatically labeled, with the correspondence between tissue segments manually determined. Each tissue part in the corrected histology image was aligned with the corresponding tissue parts in the block face image through multiscale 2D rigid registration. The aligned reference volume provided sufficient spatial information along the z-direction (anterior–posterior) for 3D segmentation and annotation of brain structures. Subsequently, 2D annotations from coronal immunofluorescent images were converted into a 3D format using a combination of automated and manual pipelines (Fig. 3*A*). Transformation parameters, including deformation fields from nonlinear registration of coronal images to block face images, were applied to 2D to provide an initial label volume with enhanced smoothness. These annotations were stacked to create labeled volumes, manually corrected for labeling errors, and neighboring slices’ labels were interpolated to yield isotropic volumes (*Materials and Methods*). A 3D mesh was generated from these isotropic label volumes using the marching cubes algorithm (54). Despite careful manual refinement with alignment and interpolation, the generated 3D mesh often exhibited irregularities along the *z*-axis. To enhance smoothness, Laplacian filters were iteratively applied to each region’s 3D mesh. While this improved visual smoothness, it occasionally led to mesh expansion and overlaps between neighboring regions. In such cases, overlapping regions were identified, and voxels in the overlapping areas were manually unassigned from one of the regions. Each region was iteratively expanded until boundaries met, and this process of smoothing and correcting was repeated until there were no overlapping regions remaining. The eLemur 3D digital atlas offers enhanced spatial comprehension of the intricate anatomy of the mouse lemur brain, thereby advancing our understanding of its structure and function (Fig. 3*B*).

### 3D Brain Cell Atlas of the Mouse Lemur.

**Fig. 4.**
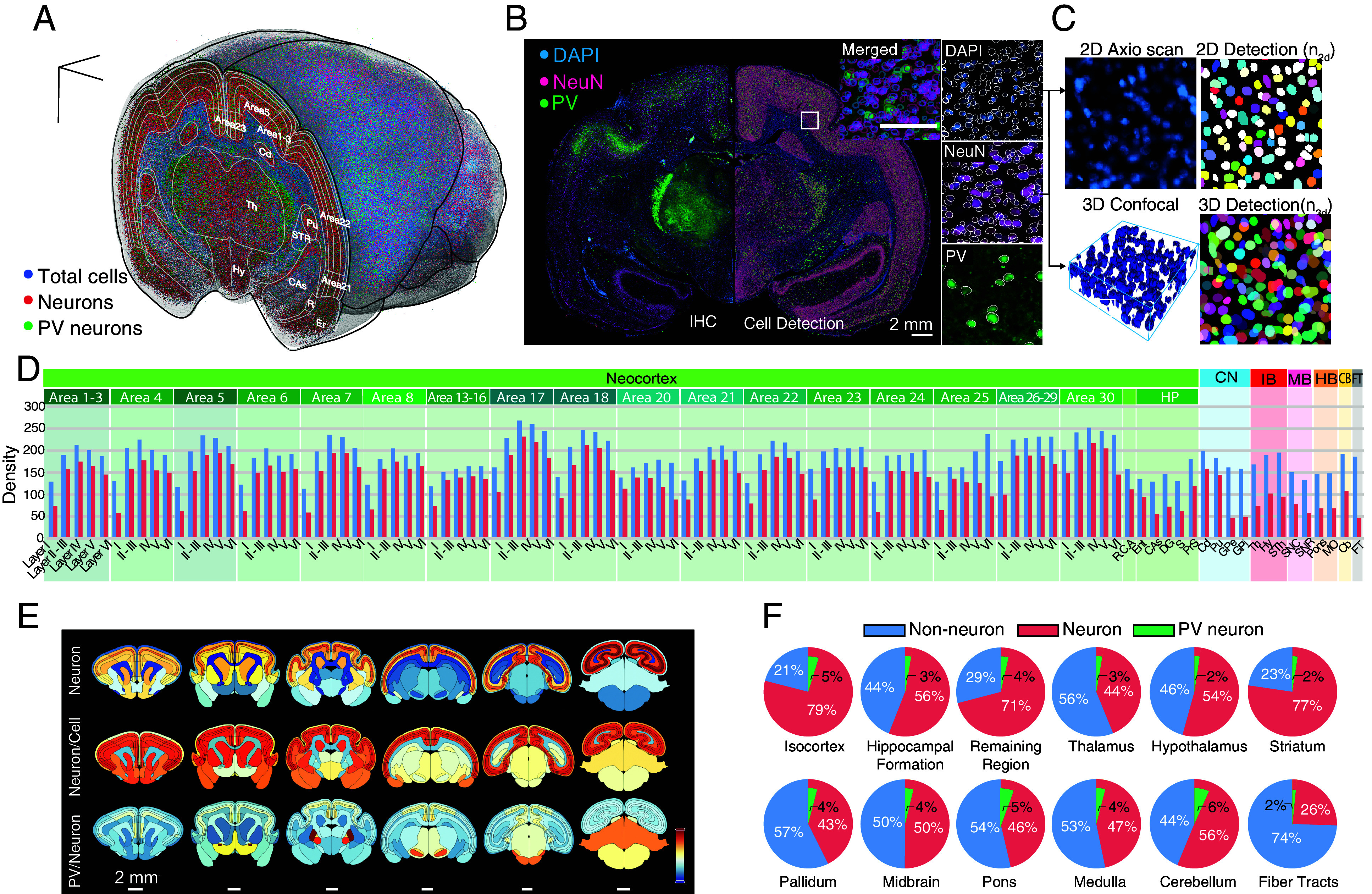
3D brain cell atlas of the mouse lemur brain. (*A*) Cell atlas displaying the topographical distributions of total cells (blue), neurons (magenta), and PV neurons (green) in 3D, labeled by DAPI, anti-NeuN, and anti-PV, respectively. (*B*) Cell detection of NeuN, PV, and DAPI-labeled cells (*Right*) alongside the original immunofluorescence image (*Left*). (*C*) 2D-imaging-based cell detection calibrated in 3D by comparing cell count ratios between 3D volumetric confocal images and 2D images. (*D*) Cell composition profiles of non-neuronal (blue) and neuronal (red) cells across brain-wide subregions, including individual cortical layers. Parameter unit: number per 100 μm^3^. (*E*) Heat map of neuronal density (max value: 231.6272; min value 33.6840), neuron/total cell ratio (max value: 1; min value: 0), and PV neuron/neuron (max value: 0.17; min value: 0) ratio across the brain. (*F*) Cell compositions of non-neuronal, neuronal, and PV neurons in twelve major brain structures. The portion of PV neurons is charted in relation to the total number of neurons in each structure.

A 3D cell atlas characterizing the distribution of cells across brain regions provides a comprehensive view of the intricate cellular architecture of the brain. Such detailed mapping is essential for deciphering both the functional and structural organization of different brain regions and serves as a fundamental reference for conducting comparative studies across different species. Utilizing digitized datasets, we conducted analysis to quantitatively map region-by-region cellular compositions of the mouse lemur brain, aiming to count total, neuronal, and PV-positive cells using DAPI, NeuN, and PV-labeled cellular signals, respectively (Fig. 4*A* and SI Appendix, Figs. S6–S8 and Table S5).

Initially, we developed an automated whole-brain cell detection algorithm with deep learning, employing semi-supervised learning to bootstrap partially annotated data to fully annotated data. After proofreading, the refined fully annotated data trained a Mask R-CNN model with a ResNeXt-101-32x8d (55) backbone for detecting cells in the entire brain. Validation of our automatic detection results against manually annotated image patches (166.4 × 166.4 μm) revealed an average precision of 83.9% and a recall rate of 93.8% for DAPI and NeuN detection (SI Appendix, Fig. S7), while for PV detection, the precision was 81.2% and the recall rate was 96.6% (SI Appendix, Fig. S8). Furthermore, we benchmarked our model against Cellpose 2.0 (56), where our method exhibited superior performance, possibly due to its reliance on larger training datasets for optimal performance. Since the automatic cell detections were conducted on 50 μm thick 2D images acquired through widefield microscopy, calibration was necessary to estimate total cell counts more accurately in 3D. By imaging subsets of small patches in cortical areas using 3D confocal microscopy and manually counting cells in volumetric images, we found consistent ratios between the number of detected cells in 3D and 2D images across regions (1.730 ± 0.024, SEM for DAPI and 1.864 ± 0.029 for NeuN) (Fig. 4 *B* and *C*). Our automated 3D anatomical template generation pipeline facilitated accurate mapping of detected cell coordinates within 2D images onto 3D reference space. Consequently, we created a 3D cell atlas of the mouse lemur brain, integrating brain-wide cell estimates of composition and distribution into our 3D atlas database (Fig. 4 *D*–*F*). Detailed 3D-calibrated numbers of the total cells, neurons, PV neurons, density, and volume of each brain structure can be found in SI Appendix, Table S5 with those of mice (50, 57).

### Development of a Web-Based Explorer of the Mouse Lemur.

**Fig. 5.**
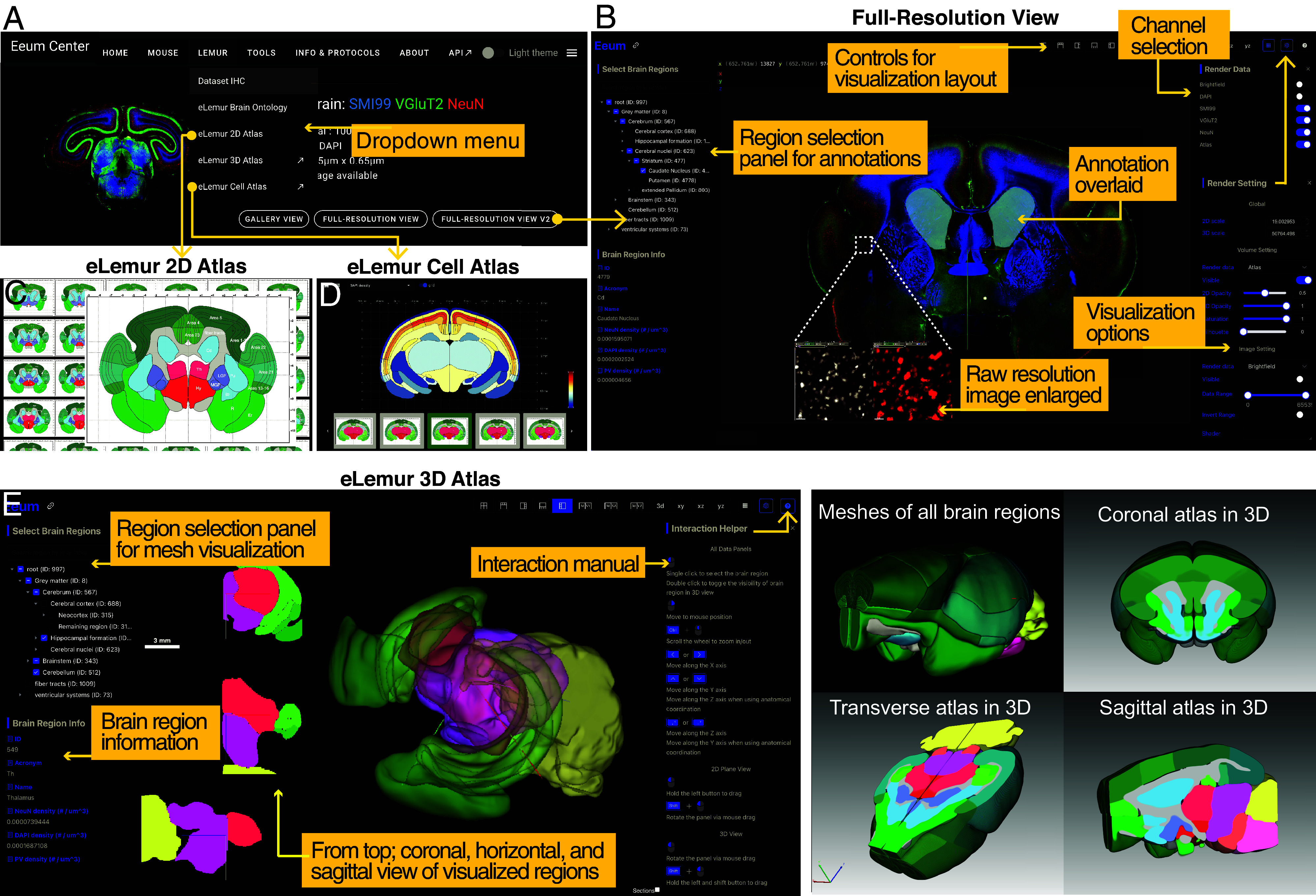
eLemur web-based explorer. (*A*) The dropdown menu from the eLemur panel allows navigation to various datasets. The Dataset IHC panel leads to the repository of immunofluorescence image datasets, featuring toolsfor antibody-specific filtering and selections, along with whole-brain gallery views for macroscopic orientation. (*B*) High-resolution slice views for detailed examination. Illustrated functionalities include annotation overlays, channel selection, and a suite of visualization settings for customized data representation. (*C*) The eLemur 2D Atlas tab presents a gallery of 2D whole-brain coronal reference images. (*D*) The eLemur Cell Atlas tab displays atlases showing the regional density of each cell type, including total cells, neurons, and PV neurons, along with ratios such as neuron/total cells and PV/total neurons. (*E*) The eLemur 3D Atlas tab provides an interactive 3D mesh visualization, allowing users to explore and comprehend the brain’s spatial organization. The interface offers flexible viewing options, an interactive manual, and detailed information about brain regions.

We have developed a comprehensive website for eLemur (Fig. 5 and Movie S3, https://eeum-brain.com/#/lemurdatasets), offering an immersive and detailed web-based platform that facilitates accessibility, collaboration, data sharing, and visualization for exploring the mouse lemur brain.

This interactive platform utilizes the Neuroglancer (58) engine to enable intricate navigation through high-resolution, multiterabyte datasets. The design of the eLemur interface aims to provide users with an unrestricted exploratory experience, effectively obviating the need for cumbersome data downloads. The main interface of the portal features a list of curated IHC datasets, with filtering options available for marker selection (Fig. 5*A*). Each dataset is displayed via a whole-brain gallery view for macroscopic orientation, alongside a full-resolution slice view that permits detailed annotation of brain regions over high-resolution images (Fig. 5*B*). The brain region selection panel facilitates precise navigation and examination of specific areas of interest. Customization of the visualization layout, image channels, and various settings is facilitated through user-operable panels, ensuring a tailored presentation of data. The reference atlas is conveyed through a 2D coronal atlas gallery with accurate physical dimensions (Fig. 5*C*), a cell atlas with regional information of cellular composition (Fig. 5*D*), and a 3D page for interactive mesh visualizations, providing a spatial understanding of structural relationships (Fig. 5*E*). Furthermore, the eLemur platform has been purposefully designed to enhance data reusability, providing researchers with tools to integrate available information into their own projects (*Materials and Methods*). This crucial feature supports ongoing studies in the field by facilitating the incorporation of existing data into future research endeavors.

## Discussion

The mouse lemur, a small primate, emerges as a promising animal model for neuroscience due to its evolutionary and genetic proximity to humans. Despite its rodent-like brain size, the species displays significant structural similarities to other primates, particularly in cortical organization, making it an appealing mode for studying various aspects of brain function, development, and evolution. Its small body size (50 to 90 g) and brain weight (approximately 2 g) also offer practical advantages, as existing laboratory tools and techniques, originally developed for mice, can be readily applied to mouse lemur research. This compatibility significantly enhances its practicality compared to other nonhuman primates like macaques and marmosets.

While rhesus monkeys (*Macaca mulatta*) and marmosets (*Callithrix jacchus*) are valuable models—rhesus monkeys for their close genetic similarity to humans and marmosets for their smaller size and reproductive traits—each species comes with its own set of considerations for researchers. For example, marmosets, though relatively small (300 to 500 g), may require modifications to laboratory setups to accommodate their specific needs. In contrast, the mouse lemur’s rodent-like body size allows researchers to more easily leverage the sophisticated tools and methodologies developed over decades for rodent studies.

The unique combination of evolutionary proximity to humans, rapid breeding cycle (with a 2-mo gestation period and large litters of 2 to 3 offspring), and quick maturation (around 1 y) make the mouse lemur an excellent model for a variety of neuroscience investigations. It is particularly well-suited for bridging the gap between rodent and primate research, facilitating translational studies. The growing mouse lemur research community, evidenced by over 300 publications in the past decade, further demonstrates its expanding role in neurobiology. Researchers are capitalizing on the mouse lemur’s versatility, applying comparable experimental paradigms across species and gaining insights into primate brain function. Notably, the mouse lemur has shown great potential for Alzheimer’s disease (AD) research, with parallels to human AD pathology, such as brain atrophy, amyloid plaques, tau pathology, and cognitive decline (59–61). Additionally, age-related sleep–wake disturbances in mouse lemurs resemble those seen in human AD patients. This expanding body of work emphasizes the mouse lemur’s value as a model for studying complex primate biology while benefiting from its shorter lifespan, ease of breeding, and adaptability to laboratory environments.

Building upon the growing recognition of the mouse lemur as a valuable model for neuroscience, we present eLemur, a platform of multiplex IHC images and atlases of the mouse lemur brain, enabling detailed exploration of its neuroanatomy at cellular and molecular levels. Our platform provides whole-brain sets of high-resolution immunostained images and 2D/3D reference atlases built through multiplex histology-based delineations of brain structures. It also integrates a 3D cell atlas, providing insights into region-by-region cellular compositions, such as the densities and spatial distributions of non-neuronal, neuronal, and PV-expressing cells across diverse brain regions. eLemur serves not only as a comprehensive platform for advancing the use of the mouse lemur as a model organism in neuroscience but also facilitates multimodal data integration, comparative analysis, and further discoveries. Our eLemur atlas represents an evolving resource, with ongoing refinements based on the latest data. For example, the hippocampus is a strong candidate for further subdivision, particularly with the distinction of the pyramidal and granular layers and the expression of VGLUT2, which helps delineate the CA2 region. The CA2 region is of particular interest due to its unique role in social memory and its distinct connectivity, making it an important area for future study (62). These features offer a promising foundation for refining subregional boundaries in future updates. Additionally, the web portal will continue to be updated based on community feedback, including adaptations to browser changes, bug fixes, and enhancements in functionality. These ongoing improvements, supported by further data collection and analysis, will ensure that eLemur remains a valuable resource for the neuroscience community.

Compared to previous Nissl-based atlases, the open access to the multiplex IHC images encourages users to further investigate interregional expressions of cellular and molecular markers, offering potential findings beyond those explored in this study. For the eLemur atlas, we aimed to achieve high-resolution IHC images and a 3D reference atlas, with 0.65 × 0.65 µm and 10 µm isotropic resolution, respectively. To our knowledge, eLemur provides the richest information on multiple marker proteins with the highest spatial resolution of any available 3D brain atlas of primates, comparable only to the Allen Mouse Brain Atlas (50). Additionally, we sought high accuracy in brain section alignment and cell counting. Using block face images collected during whole-brain sectioning, 2D-IHC images could be accurately aligned to restore the original brain section. The accuracy of cell quantification in 3D has been enhanced by calibrating 2D-based cell detection counts with 3D-based cell counts, resulting in more precise density measurements.

Furthermore, through the implementation of deep-learning methods, we showcase computer-assisted region segmentation, which significantly enhances the efficiency of annotating brain structures on microscopy images (SI Appendix, Fig. S4). In recent years, the rapid development and extensive application of image classification methods using machine learning have revolutionized the analysis of biomedical images, including fluorescent images (63–67). Despite these advancements, the segmentation of anatomical structures still relies heavily on human expertise to identify the plane of a region through comparison to preexisting literature, decide the scope of a region through putting together anatomical cues, and assign names to recognized structures. In face of such tedious procedures, our approach offers a significant advancement in segmenting images that share only partial overlapping staining features with those used in the training set through bootstrapping steps. Moreover, we have effectively employed a similar deep learning approach for cell counting and density estimation in individual brain regions, showcasing the versatility of these techniques in quantitative neuroanatomy (Fig. 4). While the current version of the eLemur atlas offers comprehensive insights into cellular compositions, including non-neuronal, neuronal, and PV-expressing cells, we acknowledge that it serves as a foundational resource for further anatomical refinements. We recognize the significant potential of leveraging cell density information to assist in more precise delineations of brain structures. However, relying solely on cell density for automatic segmentation presents notable challenges, as variations in cell density do not always align with anatomical boundaries. To achieve accurate segmentation, it is essential to integrate cell density data with additional modalities, such as molecular markers and connectivity information. This underscores the necessity of multimodal approaches in future research. As more markers and data are incorporated into the eLemur platform and as computational techniques advance, we envision the ongoing refinement of the atlas to capture finer structures with greater accuracy. Such efforts will significantly enhance the utility of the eLemur atlas, ultimately providing a more detailed and accurate representation of mouse lemur brain anatomy. Furthermore, we anticipate future research will focus on developing smarter, machine-learning-based computational algorithms that leverage cell density information for predictive delineation, similar to in silico labeling or connectivity mapping, to deepen our understanding of brain architecture through cell density-assisted delineation.

While the eLemur atlas represents a significant advancement, several challenges and limitations remain. Tissue distortions, anatomical variations, and technical constraints inherent in brain mapping techniques underscore the necessity for continued refinement and expansion of the atlas. Future research endeavors should prioritize delineating further detailed brain structures, integrating additional markers and imaging modalities, and addressing the heterogeneity of brain structures across individuals. The reliance on a single reference specimen for annotation may inadequately capture the anatomical variability present across diverse individuals and populations of mouse lemurs.

In recent decades, neuroscience research has predominantly focused on a narrow selection of species, often overlooking the vast biological diversity across the animal kingdom (8). Despite the increasing recognition of the limitations inherent in rodent models, the adoption of alternative model systems has been relatively slow. However, incorporating diversity in animal models is essential for understanding the breadth of biological mechanisms across species. The mouse lemur presents a distinct advantage in this regard. It bridges the gap between the rich array of tools and insights derived from rodent-based studies and the need for models that more closely align with human biology. As a nonhuman primate, the mouse lemur shares closer evolutionary and neurobiological similarities with humans than rodents do. Integrating the mouse lemur into neuroscience research allows scientists to explore unique evolutionary adaptations and neurobiological characteristics specific to primates, while also benefiting from the practical advantages of working with a small, easily maintainable species.

In conclusion, the mouse lemur emerges as a promising animal model in neuroscience research due to its potential to bridge the gap between existing mouse studies and nonhuman primates/humans. By embracing the biological diversity offered by the mouse lemur, researchers can expand our knowledge in neuroscience and address the complex questions underlying brain function across species. The eLemur atlas serves as a valuable resource for the neuroscience community, offering detailed insights into the neuroanatomy of the mouse lemur brain.

## Materials and Methods

### Animals.

Five mouse lemur brains (from one male and four females) born and raised in the laboratory colony of UMR 7179 (CNRS/MNHN, Brunoy, France; license approval n° A91.114.1) were used in this study. The animals were maintained in cages enriched with branches and wooden nest boxes at a standard temperature of 24 to 26 °C and relative humidity of 55%. The animals were fed with fresh fruits and a laboratory-made porridge of cereals, milk, and eggs. Water and food were available ad libitum. Animals were tested in summer-like photoperiod (14 h of light/d). The age range was 11 to 45 mo (mean ± SD 26 ± 14.697), which is considered as young to middle-aged adults (60). All animal care and experimental procedures were approved by the University of Geneva and French ethics committee “Comité d’éthique Cuvier” (authorization APAFIS#2083-2015090311335786). The delivery of the brain specimens was approved in accordance with the convention on international trade in endangered species of wild fauna and/or flora since histological experiments were partially performed in Korea (export permit FR1809100082-E and import permit ES2018-03258).

### Tissue Preparation, Block Face Imaging, Histology, and Microscopy.

Mouse lemurs were anesthetized and perfused transcardially with 0.1 M phosphate-buffered saline (PBS) and 4% paraformaldehyde in 0.1 M phosphate buffer (PFA). Brains were postfixed in 4% PFA overnight and incubated in 20% sucrose in PBS at 4 °C for cryoprotection. Brains were sectioned coronally at 50 µm thickness (approximately a total of 360 sections per brain) on a freezing microtome (Fisher Scientific HM450). During sectioning, block face images (cutting planes) of the entire brains were photographed with a complementary metal-oxide-semiconductor camera (Leica IC90 E, image size of 2592 px × 1944 px) mounted on a stereomicroscope (Leica M60).

For immunofluorescence, brain sections at a 100-µm interval were permeabilized in 0.3% Triton X-100 in tris-buffered saline (TBS) and blocked in 3% normal goat serum, 3% bovine serum albumin, and 0.3% Triton X-100 in TBS. The sections were incubated with primary antibodies overnight at 4 °C (See SI Appendix, Table S1 for the details of antibodies used). After washing, sections were incubated with secondary antibodies for 3 h at room temperature and counterstained with DAPI. Sections were mounted with mounting media (Vector Labs, H-1400). Secondary antibodies (1:1,000) used were Alexa Fluor 488 goat anti-rabbit IgG (Invitrogen, A11034), Alexa Fluor 488 goat anti-mouse IgG (Invitrogen, A11029), Alexa Fluor 555 goat anti-mouse IgG (Invitrogen, A21424), Alexa Fluor 555 goat anti-rabbit IgG (Invitrogen, A21428), Alexa Fluor 633 goat anti-guinea pig IgG (Invitrogen, A21105), and Alexa Fluor 633 goat anti-rat IgG (Invitrogen A21094).

Mice were anesthetized, and brains were perfused using the same procedures as above. Mouse brains were postfixed in 4% PFA overnight and sectioned coronally at 50 µm thickness (approximately 240 sections per brain) on a vibratome (Leica VT1200S). Brain sections containing regions of interest were permeabilized in 1% Triton X-100 in TBS and blocked in 5% normal goat serum, goat anti-mouse Fab fragments (Jackson Lab, 715-007-003) and 0.4% Triton X-100 in TBS. Identical antibodies and procedures for IHC were used as above. Widefield images were acquired using an Axioscan Z1 slide scanner (Carl Zeiss Microscopy) equipped with a 10× 0.45 NA Plan-Apochromat air lens. For cell counting analysis, confocal images were obtained at 0.54 μm depth intervals using the LSM 780 confocal microscope (Carl Zeiss Microscopy) equipped with a 40× 1.4 NA Plan Apochromat oil lens.

### Shading Correction.

A custom python script was used to apply the BaSiC algorithm (68) for flat-field estimation and shading correction on 2D-histology images. To ensure even illumination across sections within a single brain image set, we randomly extracted 20 image tiles (2,040 px × 2,040 px) from each section, combining them to estimate flat-field profile using BaSiC with default parameters. The estimated flat-field profile was then resized to match the original image size. A single flat-field profile was then used to correct shading in all image sections independently.

### 2D Block Face Alignment.

Using a custom-built MATLAB GUI, 2D histology image series were manually inspected for missing or damaged tissues and mispositioning, including flipping and rotation. Rotated or flipped images were preprocessed to center them in the correct orientation, providing better initialization for the following registration process. Tissue areas in both histology and block face images were semiautomatically labeled, with adaptive thresholding followed by manual correction. For images containing multiple disjoint tissues, correspondence between histology and block face images was determined manually by human experts. Tissue part in the histology images were aligned to the corresponding parts in the block face image via multiscale 2D rigid registration from Advanced Normalization Tools (69) in Python.

### Semiautomatic and Manual Annotation.

A custom software application with a GUI was developed to enable manual annotation of brain regions through cubic spline curves, defined by control points. Annotations were made using a computer mouse or an Apple Pencil as the input device (SI Appendix, Fig. S4C). Cubic spline curves, due to their scale-invariant nature, allowed for efficient storage and flexible editing of large-volume annotations. 13 major brain regions were manually annotated, and these data were utilized to train a deep learning segmentation network to automate the annotation of unlabeled images (SI Appendix, Fig. S4D). Due to the complex morphology of brain regions, a PointRend model (70) with a ResNeXt-101-32x8d backbone was employed. This model was trained for 192,000 iterations on four NVIDIA Tesla V100 GPUs. A learning rate initially set at 0.01 was reduced by a factor of 10 at the 48,000th, 128,000th, and 144,000th iterations. Data augmentation methods such as vertical and horizontal flips, random scaling, rotation, and channel shifts were applied. After fully annotating the major regions throughout the dataset, subregions were delineated by drawing virtual “cut lines” on the annotated major brain regions (SI Appendix, Fig. S4E). The cut lines were utilized to segment the major brain regions, and each resulting section was manually assigned the appropriate region ID, resulting in the finalized brain region annotations.

### 3D Template Atlas Generation.

To generate initial 3D template atlas volume, additional 2D nonlinear multiscale registration was performed between the binary mask images of histology and corresponding block face image and later applied to the 2D label images. Symmetric diffeomorphic registration (71) was used to interpolate 10 sections between two consecutive label images, achieving a 10 µm resolution along anterior–posterior direction. Interpolation was applied independently to each brain region. Since both initial nonlinear registration and interpolation were incomplete, interpolated regions had overlaps. In such cases, overlapping voxels were unassigned from either region, and each region was equally expanded iteratively until the boundaries touched and filled the gap. Once the 3D isotropic (10 × 10 × 10 µm) label volume with no overlapping was ready, a 3D mesh for each region was generated using the marching-cube algorithm (54). To generate a smoother isosurface, a Laplacian filter (λ = 1e−4, iteration = 100) was applied to the 3D mesh of each region. Consequential region overlapping was resolved by transforming the smoothed mesh back to the 3D volume image and overlapped voxels were fixed within voxel space. The smoothing parameters of Laplacian filters were gradually decreased each step of iterations to maintain boundary sharpness.

### MRI.

A publicly available dataset of a population averaged T2-weighted MR brain template (31) was used to annotate 19 Brodmann areas. The population averaged T2-weighted MR brain template and corresponding annotation labels were registered onto 3D block face volume via multilevel registration consisting of a series of 3D affine and nonlinear transformation using Advanced Normalization Tools (69) in Python. Cortical regions from a 2D slice of registered annotation labels were registered onto cortical areas in the corresponding eLemur slice via 2D deformable registration. The transferred annotations were manually refined to ensure the boundaries between regions lay parallel to radial direction.

### Cell Detection Model Training and Validation.

The Detectron2 framework (72) was employed to train the model for DAPI and NeuN detection. The training dataset comprised 20 images and 3,646 cell annotations. A Mask R-CNN model with a ResNeXt-101-32x8d backbone, initialized with the weights of the model pretrained on the Microsoft Common Objects in Context dataset, was utilized. This model was trained for 6,000 iterations on two NVIDIA Tesla V100 graphics processing units (GPUs) with a batch size of 6. A fixed learning rate of 0.01, along with weight decay and gradient norm clipping, was applied. Vertical and horizontal flipping and random scaling were applied for image augmentation. For PV cell detection, which exhibit a distinct appearance from DAPI and NeuN, a separate model with the same architecture was trained on a dataset containing 77 images with 775 annotations, using the same hyperparameters and procedures as the DAPI and NeuN model, but with an adjusted batch size of 8. To benchmark our model against Cellpose 2.0, we followed the hyperparameters described in the original paper (56) to fine-tune specialist Cellpose models, utilizing our two distinct training datasets for DAPI/NeuN and PV cell types. The fine-tuning process was conducted through the Cellpose 2.0 training application programming interface (API), with a learning rate of 0.1, for a total of 500 epochs. All other parameters remained at their default configurations. This training utilized a single Tesla V100 GPU equipped with 32 GB of memory.

For the validation of cell detection models, image patches of 166.4 × 166.4 μm were randomly selected from various brain regions and annotated based on consensus from six experts. The “cellpose.metrics.average_precision” function from the Cellpose 2.0 evaluation API was used to calculate average precision, recall rate, and the F1-score. Masks with an Intersection over Union of 0.5 or greater compared to ground-truth labels were considered true positives, while those below this threshold were labeled as false positives. Nondetected ground truths were classified as false negatives. A total of 29 annotated image patches were used for validating the models for DAPI and NeuN detection. The Cellpose 2.0 model’s performance was further assessed by training with 18 additional image patches, with the remaining 11 patches serving as the test set, demonstrating performance enhancement with more training data. For PV detection, nine image patches were used to assess the performance of both our model and the Cellpose 2.0 model.

### eLemur Website Development and Data Access.

The eLemur website has been developed to provide neuroscientists with necessary tools for integrating high-resolution mouse lemur brain atlas and data into their own research. Customized Vue.js components were developed to construct the platform’s user interface, while the Neuroglancer (71) engine powers the full-resolution imaging pages, offering detailed navigation. These components are open-source and 
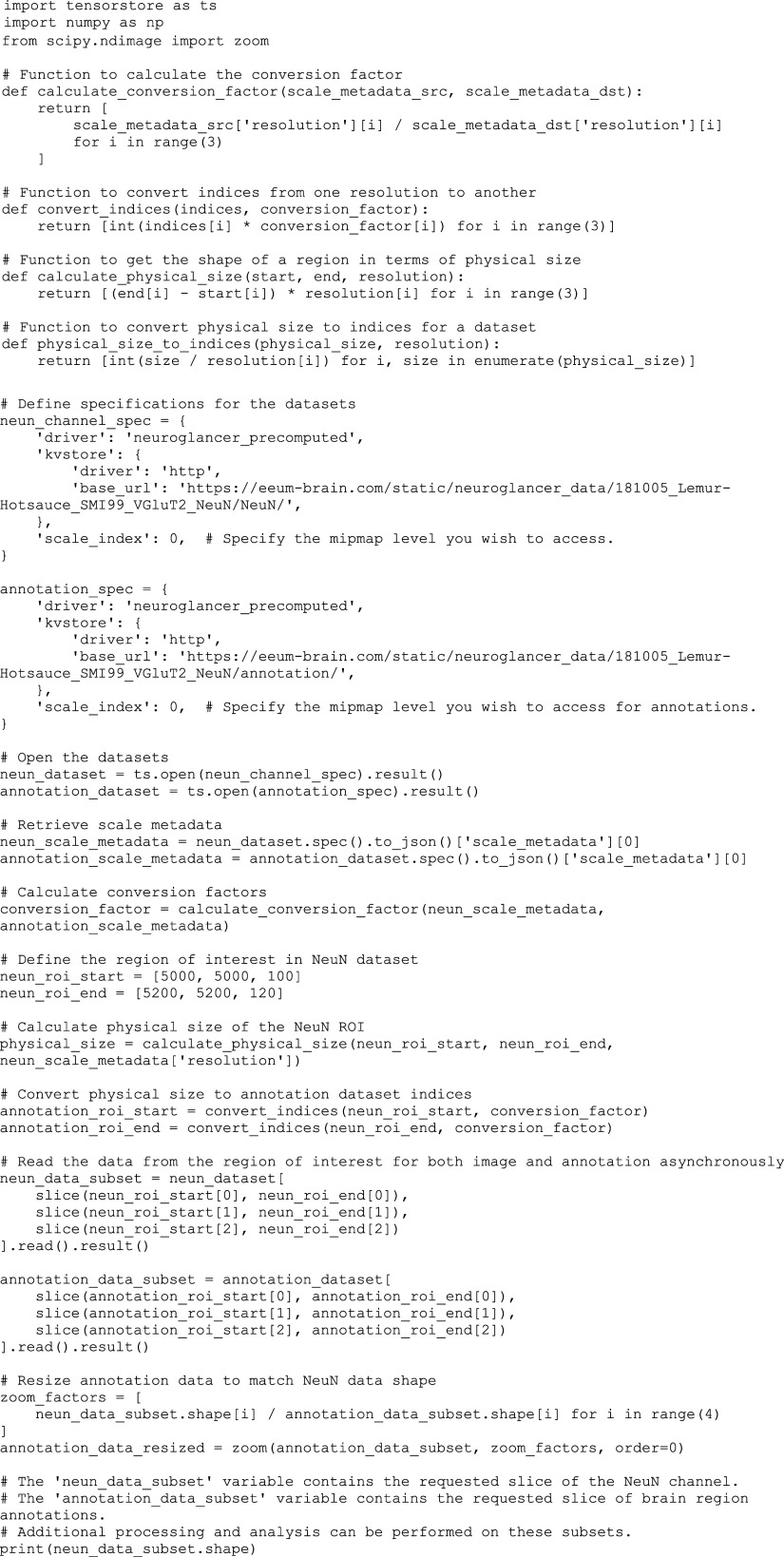
 accessible at our GitHub repository (https://github.com/feng-lab/zjbrainscience-front/tree/eeum) (73), promoting transparency and collaboration. The 3D atlas of the mouse lemur brain is available through the eLemur website and consists of three files:

(1) eLemur_3d_atlas_mesh.zip: 3D meshes of the anatomical structures
(2) eLemur_3d_atlas.mhd.zip: volumetric atlas files
(3) eLemur_3d_atlas.label: label file specifying the ID, color code, and name of each anatomical structure

Access to the eLemur image datasets is facilitated through the Tensorstore (74) library, which allows for downloading the whole dataset or a subset of the data. An example of how the Tensorstore library can be used to access and download a subset of the image data as well as its brain region annotation is provided below:

## Supplementary Material

Appendix 01 (PDF)

Movie S1. **Serial block face images with annotations**. Serial block face images of the mouse lemur brain with overlaid annotations on the left hemisphere tissue.

Movie S2. **Serial brain images immunostained with multiplex markers and annotations**. Aligned images of the mouse lemur brain immunostained with the markers such as SMI-99, VGLUT2, NeuN and DAPI. Separate fluorescent channel images of each marker are followed by the merged channels with overlaid annotations.

Movie S3. **Exploring open source data on eLemur website**. The interactive platform of eLemur aims to provide users with the IHC image datasets, 2D reference atlas, 3D interactive atlas, and cell atlas

## Data Availability

eLemur data have been deposited in GitHub (https://github.com/feng-lab/zjbrainscience-front/tree/eeum) (73). All other data are included in the article and/or supporting information.

## References

[1] D. L. Riddle, T. Blumenthal, B. J. Meyer, J. R. Priess, C. Elegans II (Cold Spring Harbor Laboratory Press, Cold Spring Harbor, NY, 1997), **vol. 33**. 21413221

[2] E. R. Kandel, The molecular biology of memory storage: A dialogue between genes and synapses. Science **294**, 1030–1038 (2001). 11691980 10.1126/science.1067020

[3] R. Menzel, The honeybee as a model for understanding the basis of cognition. Nat. Rev. Neurosci. **13**, 758–768 (2012). 23080415 10.1038/nrn3357

[4] E. Marder, Non-mammalian models for studying neural development and function. Nature **417**, 318–321 (2002). 12015611 10.1038/417318a

[5] N. Jourjine, H. E. Hoekstra, Expanding evolutionary neuroscience: Insights from comparing variation in behavior. Neuron **109**, 1084–1099 (2021). 33609484 10.1016/j.neuron.2021.02.002

[6] P. S. Katz, ‘Model organisms’ in the light of evolution. Curr. Biol. **26**, R649–R650 (2016). 27458905 10.1016/j.cub.2016.05.071

[7] C. T. Miller, M. E. Hale, H. Okano, S. Okabe, P. Mitra, Comparative principles for next-generation neuroscience. Front. Behav. Neurosci. **13**, 12 (2019). 30787871 10.3389/fnbeh.2019.00012 PMC6373779

[8] M. M. Yartsev, The emperor’s new wardrobe: Rebalancing diversity of animal models in neuroscience research. Science **358**, 466–469 (2017). 29074765 10.1126/science.aan8865

[9] A. Mathis , DeepLabCut: Markerless pose estimation of user-defined body parts with deep learning. Nat. Neurosci. **21**, 1281–1289 (2018). 30127430 10.1038/s41593-018-0209-y

[10] S. Navabpour, J. L. Kwapis, T. J. Jarome, A neuroscientist’s guide to transgenic mice and other genetic tools. Neurosci. Biobehav. Rev. **108**, 732–748 (2020). 31843544 10.1016/j.neubiorev.2019.12.013 PMC8049509

[11] S. Isik, G. Unal, Open-source software for automated rodent behavioral analysis. Front. Neurosci. **17**, 1149027 (2023). 37139530 10.3389/fnins.2023.1149027 PMC10149747

[12] Z. J. Huang, H. Zeng, Genetic approaches to neural circuits in the mouse. Neuroscience **36**, 183–215 (2013). 10.1146/annurev-neuro-062012-170307 23682658

[13] F. de Chaumont , Computerized video analysis of social interactions in mice. Nat. Methods **9**, 410–417 (2012). 22388289 10.1038/nmeth.1924

[14] T. M. Dawson, T. E. Golde, C. Lagier-Tourenne, Animal models of neurodegenerative diseases. Nat. Neurosci. **21**, 1370–1379 (2018). 30250265 10.1038/s41593-018-0236-8 PMC6615039

[15] E. A. Brenowitz, H. H. Zakon, Emerging from the bottleneck: Benefits of the comparative approach to modern neuroscience. Trends Neurosci. **38**, 273–278 (2015). 25800324 10.1016/j.tins.2015.02.008 PMC4417368

[16] P. Manger , Is 21st century neuroscience too focussed on the rat/mouse model of brain function and dysfunction? Front. Neuroanat. **2**, 5 (2008). 19127284 10.3389/neuro.05.005.2008 PMC2605402

[17] C. Ezran , The mouse lemur, a genetic model organism for primate biology, behavior, and health. Genetics **206**, 651–664 (2017). 28592502 10.1534/genetics.116.199448 PMC5499178

[18] J. Keifer, C. H. Summers, Putting the “biology” back into “neurobiology”: The strength of diversity in animal model systems for neuroscience research. Front. Syst. Neurosci. **10**, 69 (2016). 27597819 10.3389/fnsys.2016.00069 PMC4992696

[19] C. Hozer, F. Pifferi, F. Aujard, M. Perret, The biological clock in gray mouse lemur: Adaptive, evolutionary and aging considerations in an emerging non-human primate model. Front. Physiol. **10**, 1033 (2019). 31447706 10.3389/fphys.2019.01033 PMC6696974

[20] L. Roberts, Small, furry and powerful: Are mouse lemurs the next big thing in genetics? Nature **570**, 151–154 (2019). 31190019 10.1038/d41586-019-01789-0

[21] T. T. M. Consortium , Mouse lemur transcriptomic atlas elucidates primate genes, physiology, disease, and evolution. bioRxiv [Preprint] (2022). 10.1101/2022.08.06.503035 (Accessed 5 March 2024).

[22] A. Nourizonoz , EthoLoop: Automated closed-loop neuroethology in naturalistic environments. Nat. Methods **17**, 1052–1059 (2020). 32994566 10.1038/s41592-020-0961-2

[23] A. D. Yoder , Geogenetic patterns in mouse lemurs (genus Microcebus) reveal the ghosts of Madagascar’s forests past. Proc. Natl. Acad. Sci. U.S.A. **113**, 8049–8056 (2016). 27432945 10.1073/pnas.1601081113 PMC4961119

[24] C. Fichtel, J. H. der Malsburg, P. M. Kappeler, Cognitive performance is linked to fitness in a wild primate. Sci. Adv. **9**, eadf9365 (2023). 37436999 10.1126/sciadv.adf9365 PMC10337904

[25] P. Balaram, J. H. Kaas, Towards a unified scheme of cortical lamination for primary visual cortex across primates: Insights from NeuN and VGLUT2 immunoreactivity. Front. Neuroanat. **8**, 81 (2014). 25177277 10.3389/fnana.2014.00081 PMC4133926

[26] N. A. Nadkarni, S. Bougacha, C. Garin, M. Dhenain, J.-L. Picq, A 3D population-based brain atlas of the mouse lemur primate with examples of applications in aging studies and comparative anatomy. NeuroImage **185**, 85–95 (2019). 30326295 10.1016/j.neuroimage.2018.10.010

[27] J. Royo, F. Aujard, F. Pifferi, Daily torpor and sleep in a non-human primate, the gray mouse lemur (Microcebus murinus). Front. Neuroanat. **13**, 87 (2019). 31616258 10.3389/fnana.2019.00087 PMC6768945

[28] A. Langehennig-Peristenidou, D. Romero-Mujalli, T. Bergmann, M. Scheumann, Features of animal babbling in the vocal ontogeny of the gray mouse lemur (Microcebus murinus). Sci. Rep. **13**, 21384 (2023). 38049448 10.1038/s41598-023-47919-7 PMC10696017

[29] P. A. Larsen , Hybrid de novo genome assembly and centromere characterization of the gray mouse lemur (Microcebus murinus). BMC Biol. **15**, 110 (2017). 29145861 10.1186/s12915-017-0439-6 PMC5689209

[30] N. Bons, S. Silhol, V. Barbié, N. Mestre-Francés, D. Albe-Fessard, A stereotaxic atlas of the grey lesser mouse lemur brain (Microcebus murinus). Brain Res. Bull. **46**, 1–173 (1998). 9639030 10.1016/s0361-9230(97)00458-9

[31] N. A. Nadkarni, S. Bougacha, C. Garin, M. Dhenain, J.-L. Picq, Digital templates and brain atlas dataset for the mouse lemur primate. Data Brief **21**, 1178–1185 (2018). 30456231 10.1016/j.dib.2018.10.067 PMC6230976

[32] S. F. Grieco, T. C. Holmes, X. Xu, Probing neural circuit mechanisms in Alzheimer’s disease using novel technologies. Mol. Psychiatry **28**, 4407–4420 (2023). 36959497 10.1038/s41380-023-02018-x PMC10827671

[33] K. G. Schilling , A web-based atlas combining MRI and histology of the squirrel monkey brain. Neuroinformatics **17**, 131–145 (2019). 30006920 10.1007/s12021-018-9391-z PMC6330248

[34] T. M. Preuss, S. P. Wise, Evolution of prefrontal cortex. Neuropsychopharmacology **47**, 3–19 (2022). 34363014 10.1038/s41386-021-01076-5 PMC8617185

[35] M. K. L. Baldwin, J. A. Bourne, Evolution of nervous systems. Vis. Syst. **3**, 165–185 (2017), 10.1016/b978-0-12-804042-3.00081-6.

[36] B. W. Balleine, J. P. O’Doherty, Human and rodent homologies in action control: Corticostriatal determinants of goal-directed and habitual action. Neuropsychopharmacology **35**, 48–69 (2010). 19776734 10.1038/npp.2009.131 PMC3055420

[37] L. Puelles, T. Stühmer, J. L. R. Rubenstein, C. Diaz, Critical test of the assumption that the hypothalamic entopeduncular nucleus of rodents is homologous with the primate internal pallidum. J. Comp. Neurol. **531**, 1715–1750 (2023). 37695031 10.1002/cne.25536 PMC11418882

[38] J. L. Lanciego, N. Luquin, J. A. Obeso, Functional neuroanatomy of the basal ganglia. Cold Spring Harb. Perspect. Med. **2**, a009621 (2012). 23071379 10.1101/cshperspect.a009621 PMC3543080

[39] M. W. Miller, B. A. Vogt, Direct connections of rat visual cortex with sensory, motor, and association cortices. J. Comp. Neurol. **226**, 184–202 (1984). 6736299 10.1002/cne.902260204

[40] S. Ding , Comprehensive cellular-resolution atlas of the adult human brain. J. Comp. Neurol. **524**, 3127–3481 (2016). 27418273 10.1002/cne.24080 PMC5054943

[41] G. Paxinos, K. B. J. Franklin, Paxinos and Franklin’s the Mouse Brain in Stereotaxic Coordinates (Elsevier Science, 2012).

[42] L. Gao , Single-neuron projectome of mouse prefrontal cortex. Nat. Neurosci. **25**, 515–529 (2022). 35361973 10.1038/s41593-022-01041-5

[43] K. D. Harris, G. M. G. Shepherd, The neocortical circuit: Themes and variations. Nat. Neurosci. **18**, 170–181 (2015). 25622573 10.1038/nn.3917 PMC4889215

[44] A. Bernard , Transcriptional architecture of the primate neocortex. Neuron **73**, 1083–1099 (2012). 22445337 10.1016/j.neuron.2012.03.002 PMC3628746

[45] N. L. Jorstad , Transcriptomic cytoarchitecture reveals principles of human neocortex organization. Science **382**, eadf6812 (2023). 37824655 10.1126/science.adf6812 PMC11687949

[46] M. Nahmani, A. Erisir, VGluT2 immunochemistry identifies thalamocortical terminals in layer 4 of adult and developing visual cortex. J. Comp. Neurol. **484**, 458–473 (2005). 15770654 10.1002/cne.20505

[47] V. Garcia-Marin, T. H. Ahmed, Y. C. Afzal, M. J. Hawken, Distribution of vesicular glutamate transporter 2 (VGluT2) in the primary visual cortex of the macaque and human. J. Comp. Neurol. **521**, 130–151 (2013). 22684983 10.1002/cne.23165 PMC4001706

[48] B. A. Strange, M. P. Witter, E. S. Lein, E. I. Moser, Functional organization of the hippocampal longitudinal axis. Nat. Rev. Neurosci. **15**, 655–669 (2014). 25234264 10.1038/nrn3785

[49] D. Milardi , The cortico-basal ganglia-cerebellar network: Past, present and future perspectives. Front. Syst. Neurosci. **13**, 61 (2019). 31736719 10.3389/fnsys.2019.00061 PMC6831548

[50] Q. Wang , The allen mouse brain common coordinate framework: A 3D reference atlas. Cell **181**, 936–953.e20 (2020). 32386544 10.1016/j.cell.2020.04.007 PMC8152789

[51] D. M. Bowden, E. Song, J. Kosheleva, M. F. Dubach, NeuroNames: An ontology for the braininfo portal to neuroscience on the web. Neuroinformatics **10**, 97–114 (2012). 21789500 10.1007/s12021-011-9128-8 PMC3247656

[52] L. W. Swanson, Brain maps 4.0—Structure of the rat brain: An open access atlas with global nervous system nomenclature ontology and flatmaps. J. Comp. Neurol. **526**, 935–943 (2018). 29277900 10.1002/cne.24381 PMC5851017

[53] H. W. Dong, The Allen Reference Atlas: A Digital Color Brain Atlas of the C57Bl/6J Male Mouse (John Wiley and Sons, 2008).

[54] W. E. Lorensen, H. E. Cline, Marching cubes: A high resolution 3D surface construction algorithm. ACM SIGGRAPH Comput. Graph. **21**, 163–169 (1987).

[55] S. Xie, R. Girshick, P. Dollár, Z. Tu, K. He, Aggregated residual transformations for deep neural networks. arxiv [Preprint] (2016). 10.48550/arxiv.1611.05431 (Accessed 16 August 2021).

[56] M. Pachitariu, C. Stringer, Cellpose 2.0: How to train your own model. Nat. Methods **19**, 1634–1641 (2022). 36344832 10.1038/s41592-022-01663-4 PMC9718665

[57] C. Erö, M.-O. Gewaltig, D. Keller, H. Markram, A cell atlas for the mouse brain. Front. Neuroinf. **12**, 84 (2018). 10.3389/fninf.2018.00084 PMC6280067 30546301

[58] E. Perlman, Visualizing and interacting with large imaging data. Microsc. Microanal. **25**, 1374–1375 (2019).

[59] N. Bons, F. Rieger, D. Prudhomme, A. Fisher, K.-H. Krause, *Microcebus murinus*: A useful primate model for human cerebral aging and Alzheimer’s disease? Genes Brain Behav. **5**, 120–130 (2006). 16507003 10.1111/j.1601-183X.2005.00149.x

[60] S. Languille , The grey mouse lemur: A non-human primate model for ageing studies. Ageing Res. Rev. **11**, 150–162 (2012). 21802530 10.1016/j.arr.2011.07.001

[61] F. Pifferi, J. Epelbaum, F. Aujard, Strengths and weaknesses of the gray mouse lemur (*Microcebus murinus*) as a model for the behavioral and psychological symptoms and neuropsychiatric symptoms of dementia. Front. Pharmacol. **10**, 1291 (2019). 31736761 10.3389/fphar.2019.01291 PMC6833941

[62] F. L. Hitti, S. A. Siegelbaum, The hippocampal CA2 region is essential for social memory. Nature **508**, 88–92 (2014). 24572357 10.1038/nature13028 PMC4000264

[63] C. Tan , DeepBrainSeg: Automated brain region segmentation for micro-optical images with a convolutional neural network. Front. Neurosci. **14**, 179 (2020). 32265621 10.3389/fnins.2020.00179 PMC7099146

[64] Z. Li , D-LMBmap: A fully automated deep-learning pipeline for whole-brain profiling of neural circuitry. Nat. Methods **20**, 1593–1604 (2023). 37770711 10.1038/s41592-023-01998-6 PMC10555838

[65] X. Wang , Bi-channel image registration and deep-learning segmentation (BIRDS) for efficient, versatile 3D mapping of mouse brain. Elife **10**, e63455 (2021). 33459255 10.7554/eLife.63455 PMC7840180

[66] Y. Amitay , Cell Sighter: A neural network to classify cells in highly multiplexed images. Nat. Commun. **14**, 4302 (2023). 37463931 10.1038/s41467-023-40066-7 PMC10354029

[67] K. Yao, N. D. Rochman, S. X. Sun, Cell type classification and unsupervised morphological phenotyping from low-resolution images using deep learning. Sci. Rep. **9**, 13467 (2019). 31530889 10.1038/s41598-019-50010-9 PMC6749053

[68] T. Peng , A BaSiC tool for background and shading correction of optical microscopy images. Nat. Commun. **8**, 14836 (2017). 28594001 10.1038/ncomms14836 PMC5472168

[69] N. J. Tustison , The ANTsX ecosystem for quantitative biological and medical imaging. Sci. Rep. **11**, 9068 (2021). 33907199 10.1038/s41598-021-87564-6 PMC8079440

[70] A. Kirillov, Y. Wu, K. He, R. Girshick, “PointRend: Image segmentation as rendering” in 2020 IEEE/CVF Conference on Computer Vision and Pattern Recognition (CVPR) (IEEE, Seattle, WA, 2020), pp. 9796–9805.

[71] B. B. Avants, C. L. Epstein, M. Grossman, J. C. Gee, Symmetric diffeomorphic image registration with cross-correlation: Evaluating automated labeling of elderly and neurodegenerative brain. Med. Image Anal. **12**, 26–41 (2008). 17659998 10.1016/j.media.2007.06.004 PMC2276735

[72] Y. Wu, A. Kirillov, F. Massa, W.-Y. Lo, R. Girshick, Detectron2. Github (2019). https://github.com/facebookresearch/detectron2. Accessed 16 August 2021.

[73] W. Wu, C. Zhang, Q. Han, L. Feng, zjbrainscience-front. Github. https://github.com/feng-lab/zjbrainscience-front/tree/eeum. Deposited 30 April 2024.

[74] TensorStore, Github. TensorStore (2020). https://github.com/google/tensorstore. Accessed 26 April 2024.

