# Supplementary material for: eLemur: A cellular-resolution 3D atlas of the mouse lemur brain
Source: Proc Natl Acad Sci U S A. 2024 Dec 4;121(50):e2413687121. doi: 10.1073/pnas.2413687121 (PMC11648901; doi:10.1073/pnas.2413687121)
Supplement: Supplementary file 1 — Appendix 01 (PDF) [file pnas.2413687121.sapp.pdf]

PNAS

Supporting Information for

eLemur: A cellular-resolution 3D atlas of the mouse lemur brain

Hyungju Jeon[1†], Jiwon Kim[1,2†], Jayoung Kim[1,2†], Yoon Kyoung Choi[1,3], Chun Lum Andy Ho[4], Fabien Pifferi[5], Daniel Huber[4], Linqing Feng[1*], and Jinhyun Kim[1,2,3,6*]

[1]Brain Science Institute, Korea Institute of Science and Technology (KIST), Seoul, South Korea
[2]Division of Bio-Medical Science & Technology, KIST-School, University of Science and Technology, Seoul, South Korea
[3]Department of Computer Science and Engineering, Korea University, Seoul, South Korea
[4]University of Geneva, Department of Basic Neurosciences, Geneva, Switzerland.
[5]Musée National d'Histoire Naturelle, Adaptive Mechanisms and Evolution, UMR7179—CNRS, Paris, France
[6]KIST-SKKU Brain Research Center, SKKU Institute for Convergence, Sungkyunkwan University, Suwon, South Korea
[†]These authors contributed equally to this work

***Correspondence:** Jinhyun Kim, Brain Science Institute at the Korea Institute of Science & Technology (KIST), 39-1 Hawolgokdong, Seongbukgu, Seoul 02792, South Korea; phone: +82-2-958-7225, jinnykim@me.com; Linqing Feng, 11A-401-7 Zhejiang Lab, Kechuang Avenue, Zhongtai Sub-District, Yuhang District, Hangzhou, Zhejiang Province, 311121 China, flq@live.com
Email: Jinhyun Kim jinnykim@me.com; Linqing Feng flq@live.com

This PDF file includes:

Figures S1 to S8
Tables S1 to S5
Legends for Movies S1 to S3
SI References

Other supporting materials for this manuscript include the following:

Movies S1 to S3

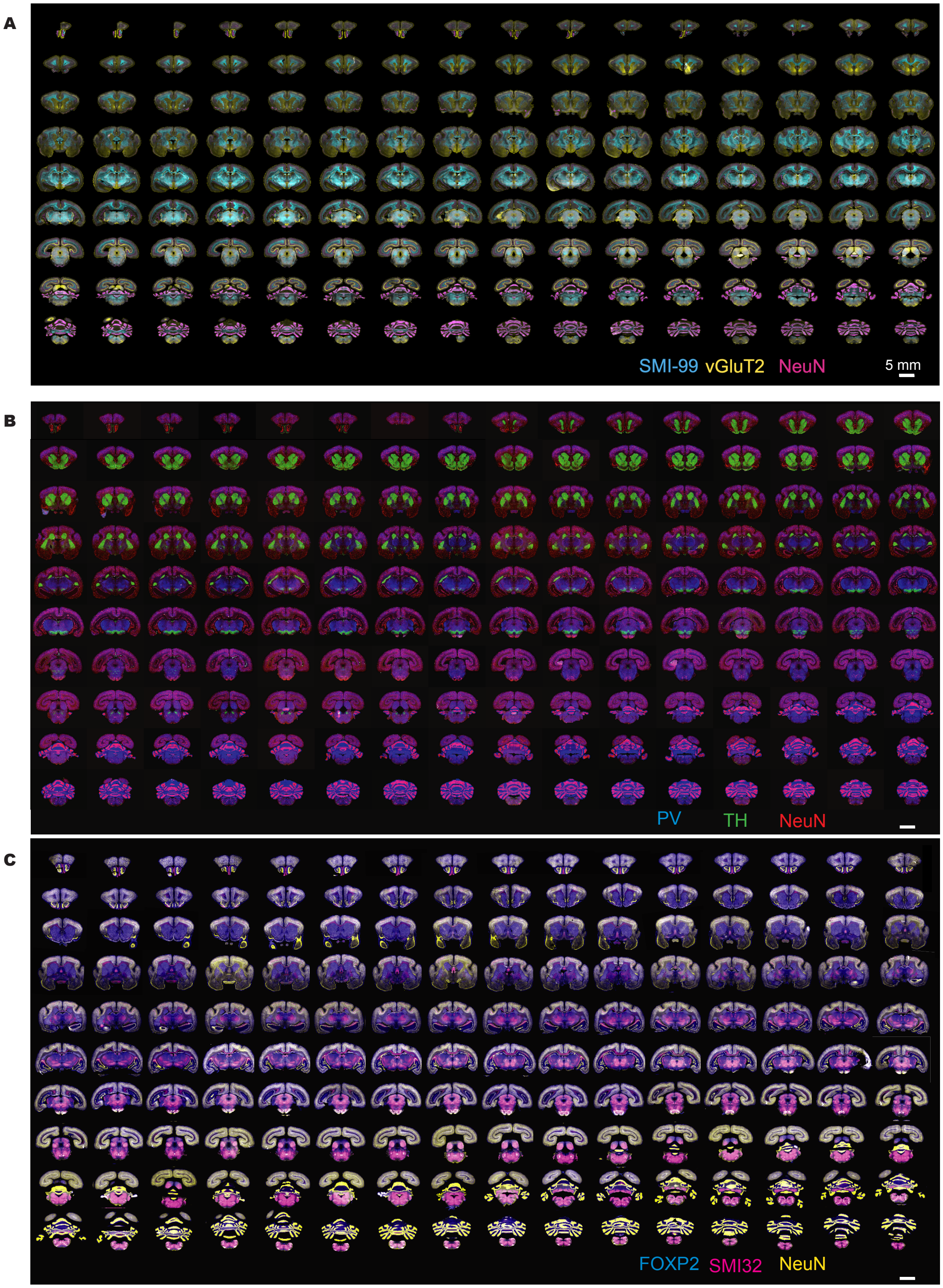

Fig. S1. Whole-brain immunofluorescence images of the mouse lemur
Gallery view of whole-brain IHC sets of the mouse lemur brain stained with **A)** SMI-99, VGLUT2, NeuN, **(B)** PV, TH, NeuN, and **(C)** FOXP2, SMI-32, and NeuN.

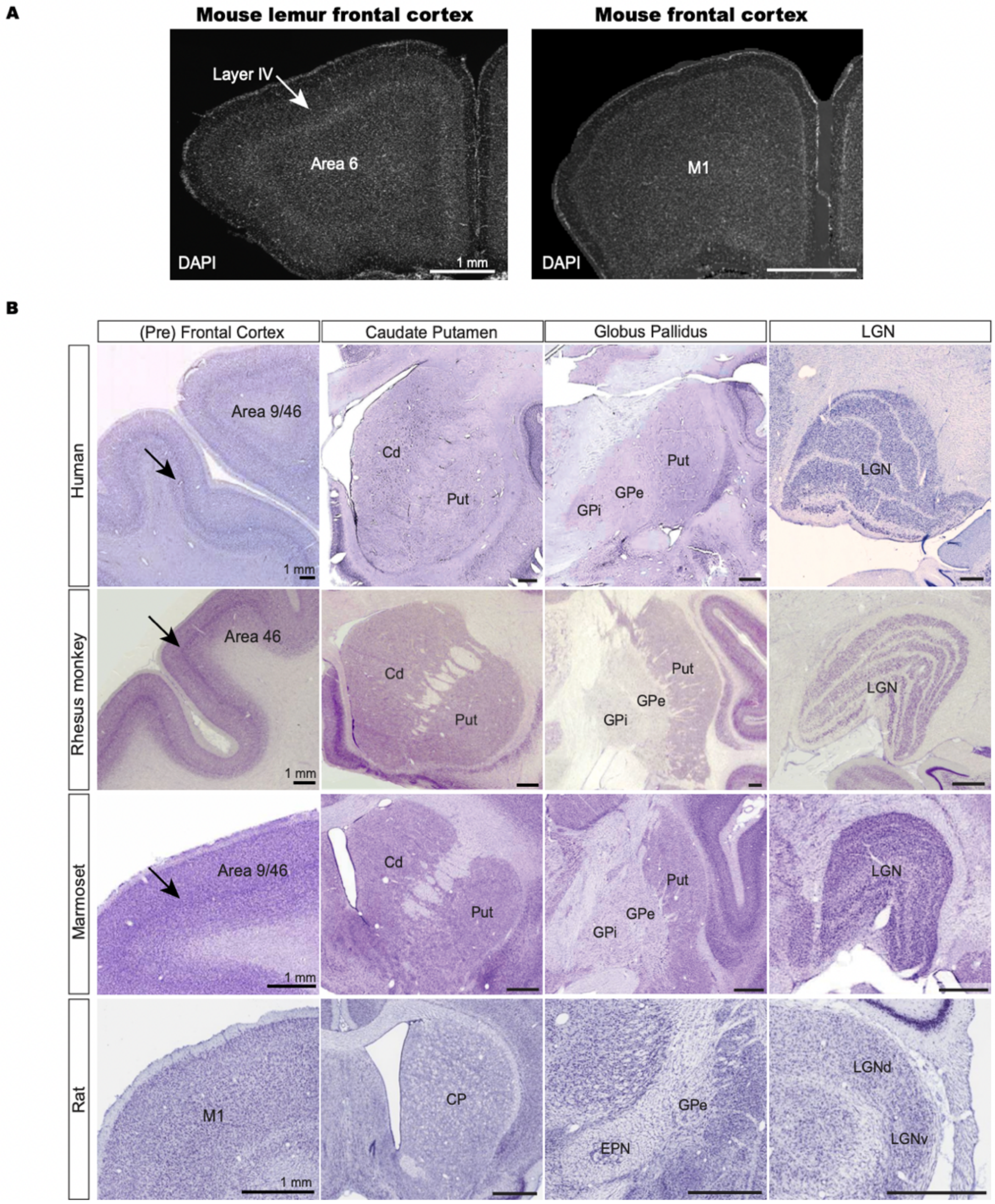

Fig. S2. Comparative analysis of the frontal cortex, basal ganglia, and LGN across species
(A) Comparison of the frontal cortex in the mouse lemur and mouse. The mouse lemur cortex shows a distinct granular layer IV that indicates a granular frontal cortex, a feature shared by the primate family. The mouse frontal cortex, on the other hand, is agranular.

(B) Comparison of the frontal cortex, basal ganglia, and LGN across species from previously published Nissl staining-based atlases. Human data were extracted from the Allen Reference Atlas, while stainings for rhesus monkey and rat were sourced from Brainmaps.org. Marmoset data was extracted from Palazzi & Bordier (2008)[1]. Primates display a granular cortex with a well-defined layer IV, marked with an arrow. The primate basal ganglia also exhibit distinct caudate and putamen segments separated by a fiber track, alongside a layered structure of the LGN. Conversely, in rodents, organization of the cortex, basal ganglia and LGN in rats differs from that in primates (also see **Figure 1D**).

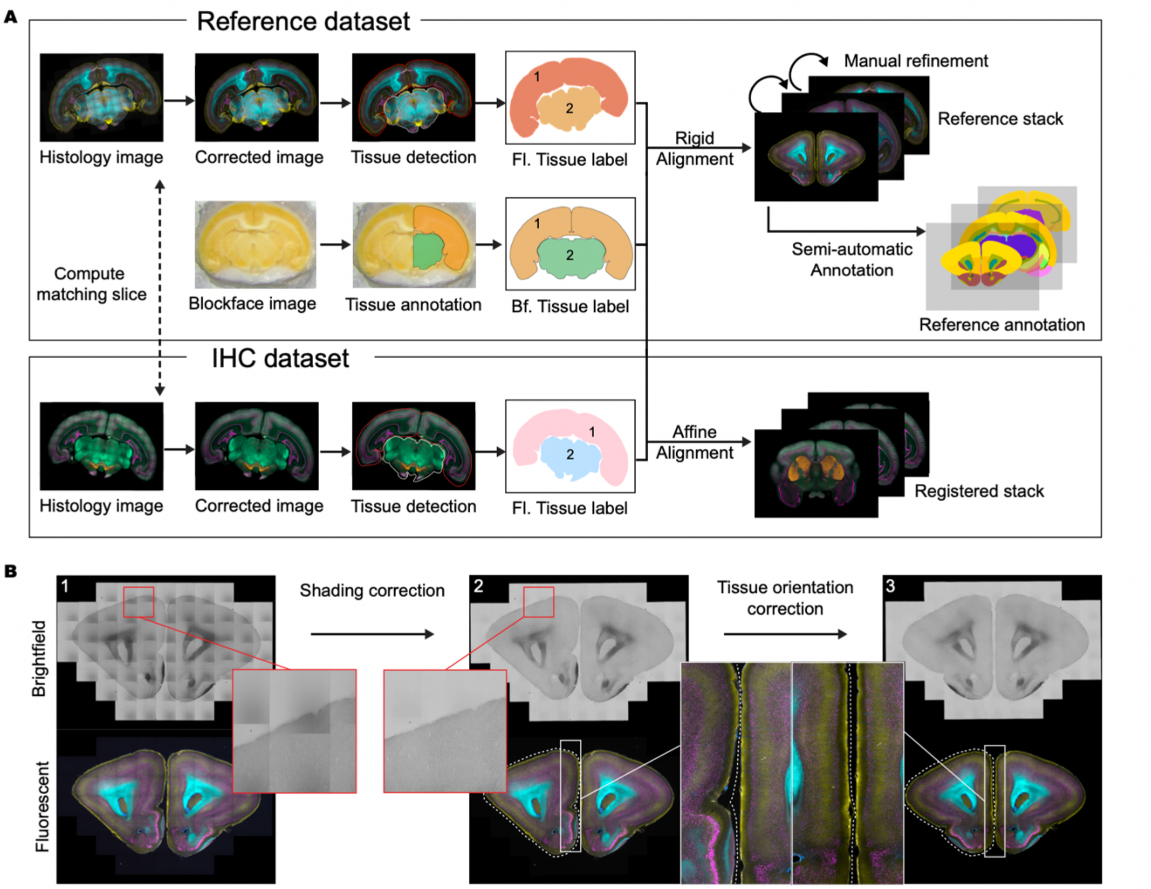

Fig. S3. Image processing pipeline for the immunofluorescent image dataset
(A) The flowchart outlines the steps involved in processing and aligning the reference and other immunohistochemistry (IHC) datasets. Immunofluorescent images first undergo correction and alignment to the corresponding block face image. Subsequently, semi-automatic region annotation is conducted. To minimize artifacts arising from deformation, only rigid transformations (rotation and translation) are applied to the reference dataset.
(B) A coronal view of a brain slice showing three stages of image processing: The original image before any correction procedures (left), the image after shading correction to enhance clarity and remove irregular illumination (middle), and the final image after undergoing repair procedures to address any remaining artifacts or imperfections (right).

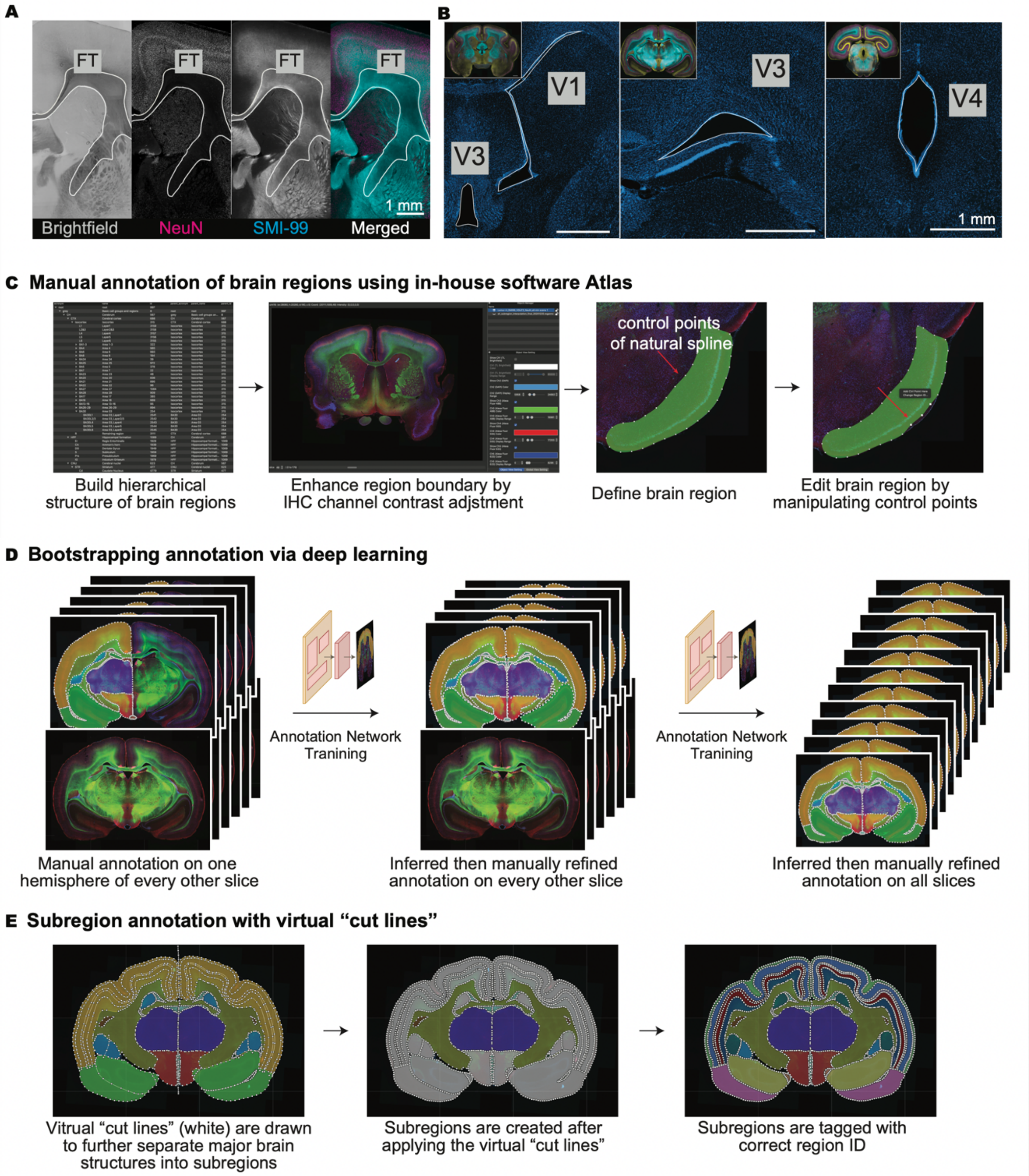

Fig. S4. Annotation of brain structures
(A, B) Fiber tracks and ventricles are delineated using multiplex immunosignals, complemented by brightfield contrast images.
(C) Manual annotation of brain regions was performed using an in-house annotation software featuring a Graphical User Interface (GUI). Brain regions were delineated with closed natural cubic splines.
(D) Annotation bootstrapping through deep learning involved training two deep region segmentation networks to propagate partial annotations across the entire stack.
(E) Subregion annotation was achieved using virtual "cut lines" to define smaller subregions by separating major brain regions.

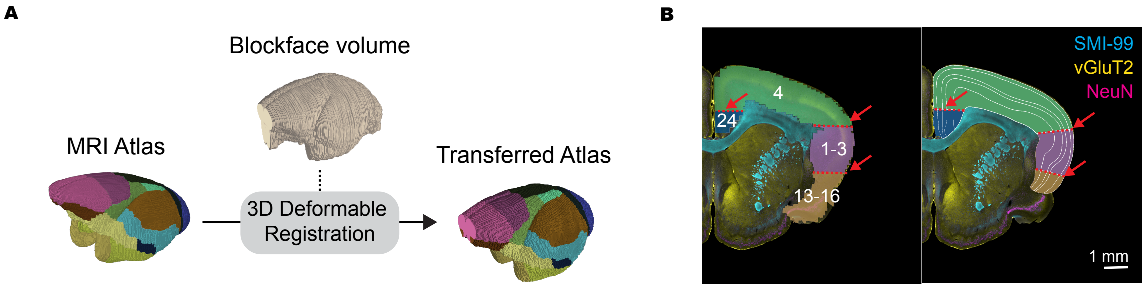

Fig. S5. Integration of the MRI-based atlas into 2D immunofluorescence images
(A) Flowchart of the population averaged T2-weighted MRI atlas registration to the block face volumetric image**.**
(B) Slice-by-Slice manual correction and refinement of registered MRI atlas. Deformed atlas is overlaid on to corresponding IHC slices and the boundaries between Brodmann area (red) are refined to 1) match cellular architectures and 2) be aligned parallel to the radial direction.

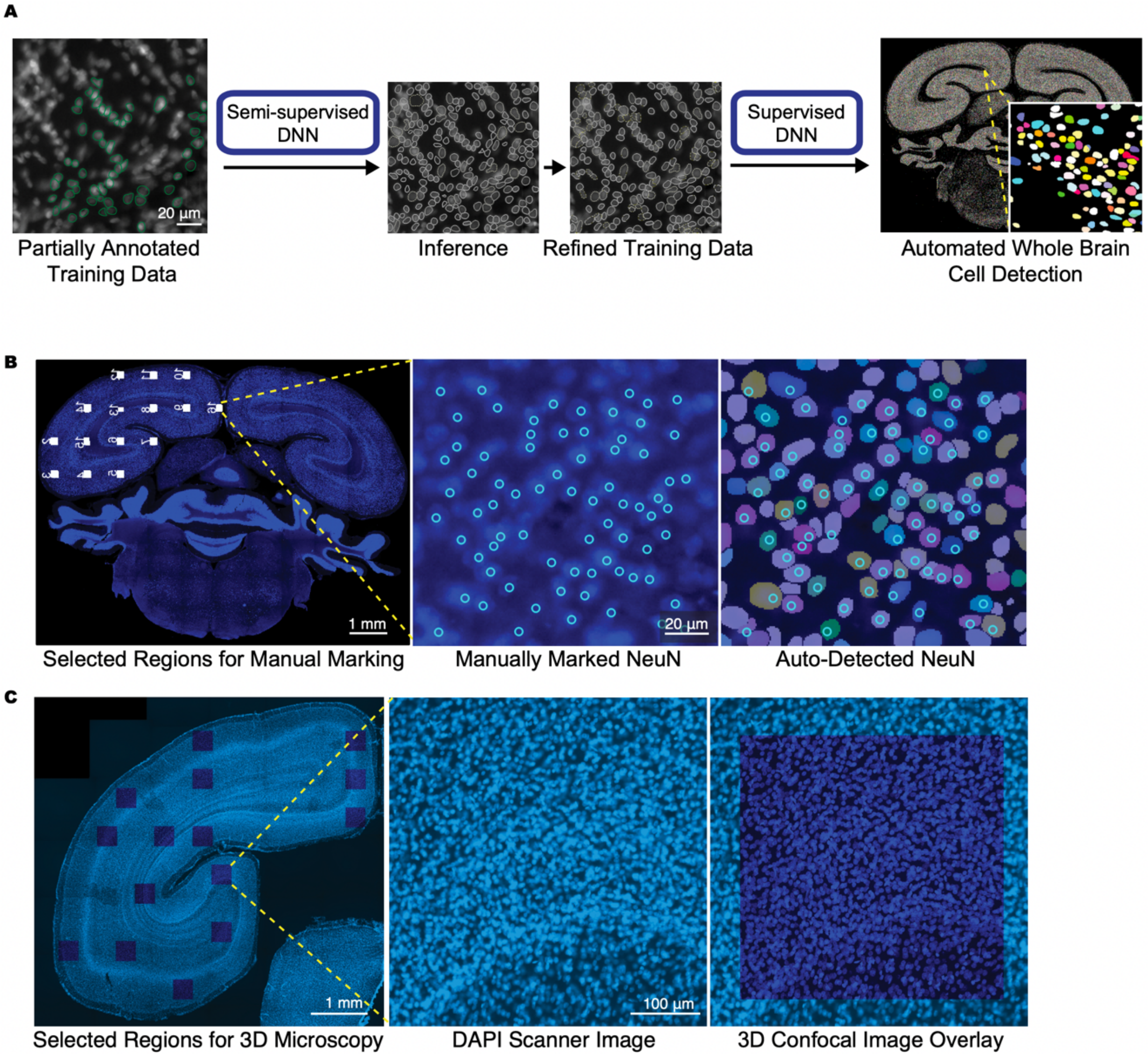

Fig. S6. Automated whole-brain cell detection algorithm
(A) Workflow of automated whole-brain cell detection algorithm based on partially annotated data. **(B)** Validation of automatic NeuN-positive neuron detection results. Small image patch is sampled uniformly within the slice (left). For each patch, NeuN cells are manually annotated by human experts (center), and automatic detection results are validated against manual detections (right). **(C)** Validation of automatic 2D DAPI-positive cell detection results against 3D confocal cell counts. Small image column is sampled from 3D confocal image and corresponding image patch from 2D IHC slice is determined (left). Detection results from 2D slice and compared and validated with 3D confocal image (center and right).

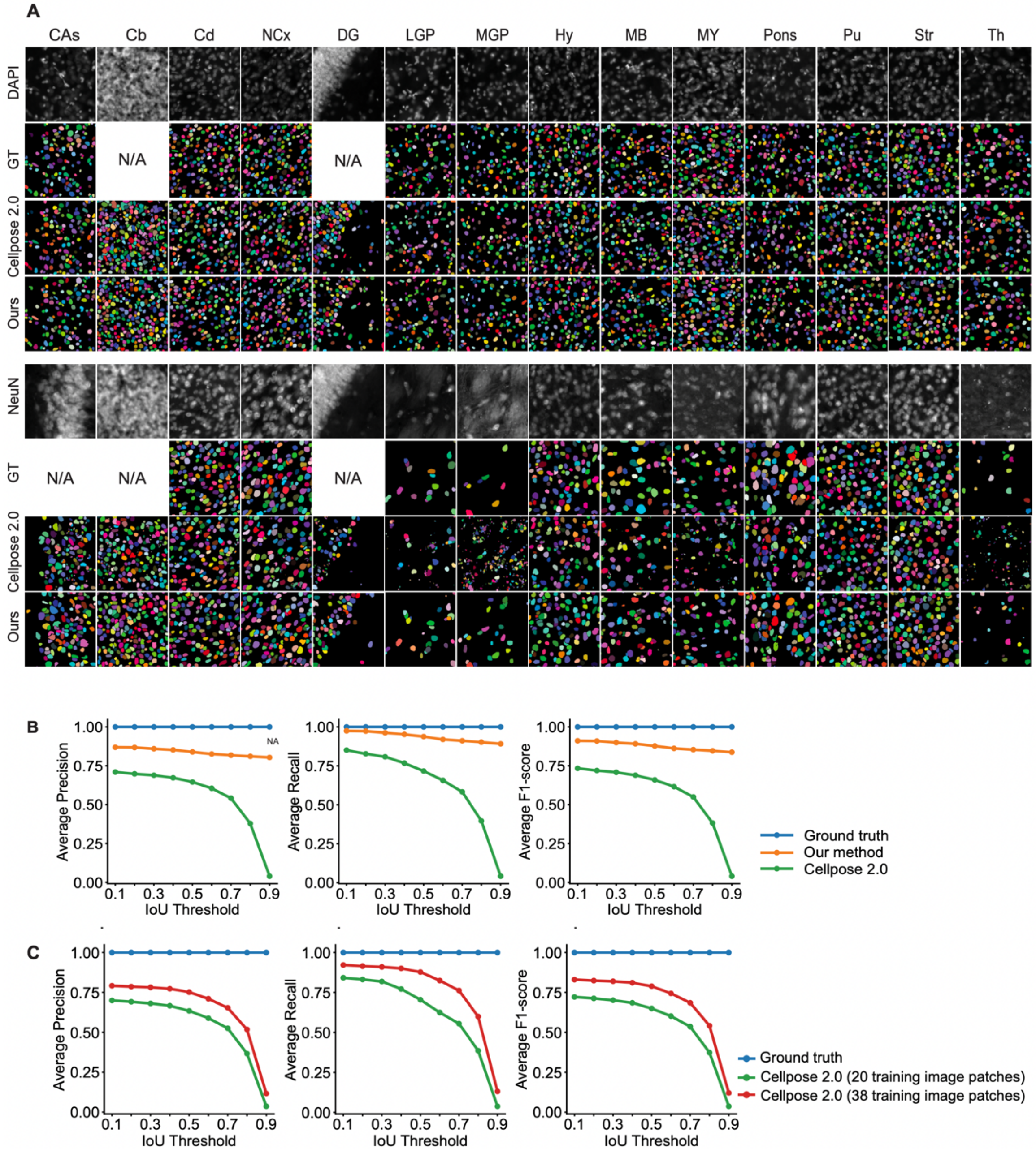

Fig. S7. Validation of DAPI and NeuN detection algorithm
(A) Random selected image patches from various brain regions, showing consensus annotations from six experts as “ground-truth (GT)”, detection results from Cellpose 2.0, and our method.
(B) Average precision, recall, and F1-score for different cell detection methods, demonstrating that our method outperforms Cellpose 2.0.
(C) Performance enhancement of Cellpose 2.0 with more training datasets.

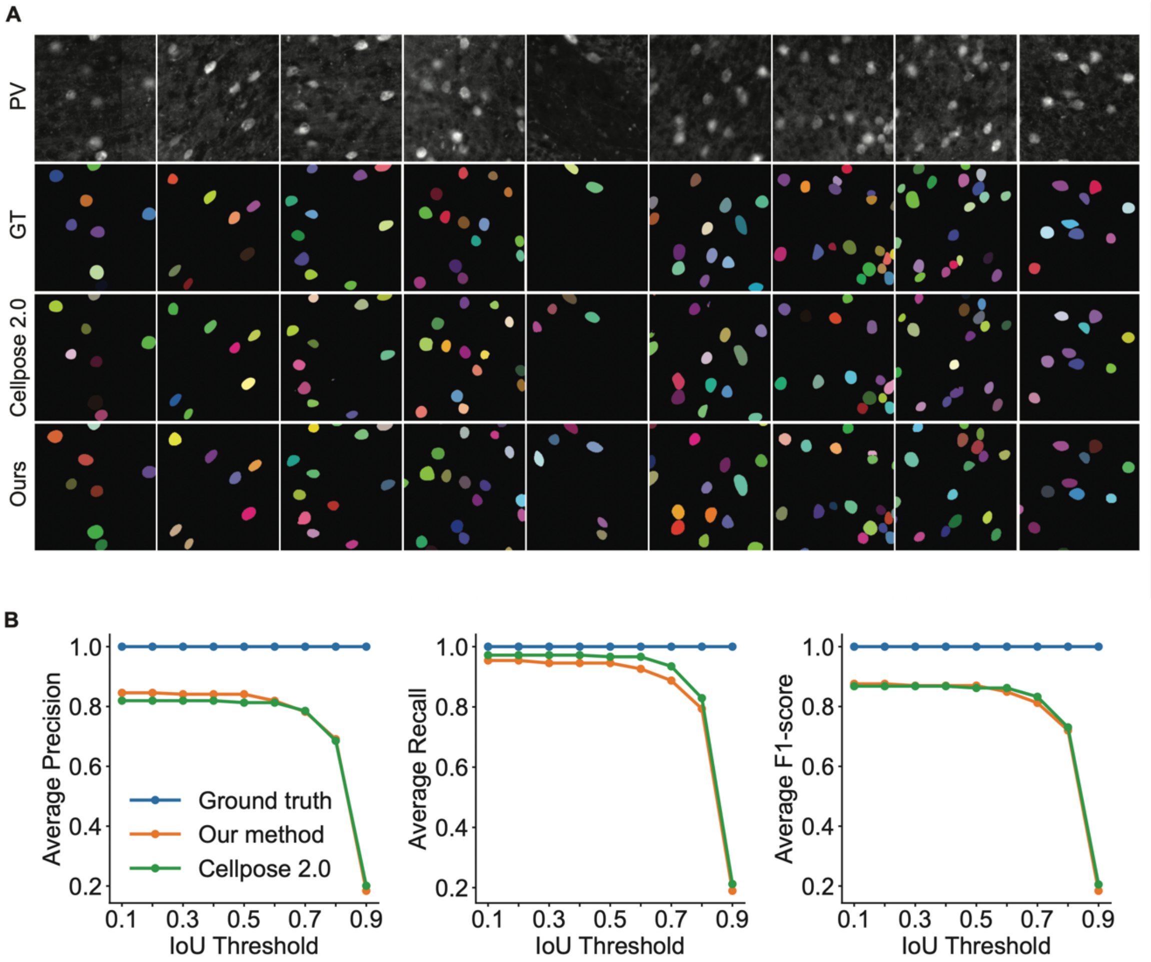

Fig. S8. Validation of PV detection algorithm
(A) Random selected image patches from the PV channel, showing manual annotations, detection results from Cellpose 2.0, and our method.
(B) Average precision, recall, and F1-score for different cell detection methods.

Table S1. Primary antibody information

Stain	Target	Host	Vendor	Cat. No.	RRID	Dilution
NeuN	Neuronal nuclear antigen	guinea pig	Millipore	ABN90P	AB_2341095	1:1000
FOXP2	Forkhead box protein P2	rabbit	Abcam	AB16046	AB_2107107	1:1000
PV	Parvalbumin	mouse	Swant	235	AB_10000343	1:1000
TH	Tyrosine hydroxylase	rabbit	Millipore	AB152	AB_390204	1:1000
SMI-99	Myelin basic protein	mouse	BioLegend	808401	AB_2564741	1:1000
SMI-32	Neurofilament H	mouse	BioLegend	801702	AB_2715852	1:1000
vGluT2	Vesicular glutamate transporter 2	rabbit	Synaptic Systems	135403	AB_887883	1:1000

Information on the primary antibodies used to generate mouse lemur whole-brain immunohistochemistry (IHC) sets. The antibodies were selected based on their compatibility with mouse lemur brain tissues after initial testing on mouse brain tissues.

Table S2. Mouse lemur brain region nomenclature

eLemur		Bons		NeuroNames		Allen Mouse	
Name	Abbr.	Name	Abbr.	Name	Abbr.	Name	Abbr.
grey	grey						
Cerebrum	CH			Cerebrum		Cerebrum	CH
Cerebral cortex	Cx			Cerebral cortex	Cx	Cerebral cortex	CTX
Neocortex	NCx			Neocortex		Isocortex	ISO
Hippocampal formation	HiF	Hippocampus	H	Hippocampal formation	HiF	Hippocampal formation	HPF
Dentate gyrus	DG	Gyrus Dentatus	GD	Dentate gyrus	DG	Dentate gyrus	DG
CA fields	CAs			CA fields	CAs	Ammon's horn	CA
entorhinal area	Ent	Regio entorhinalis	Er	Regio entorhinalis		Entorhinal area	ENT
Subiculum	S	Subiculum	S	Subiculum	S	Subiculum	SUB
Presubiculum	PrS	Regio praesubicularis	Prs	Presubiculum	PrS	Presubiculum	PRE
Cerebral nuclei	CNu					Cerebral nuclei	CNU
Striatum	Str			Striatum	Str	Striatum	STR
Caudate nucleus	Cd	Nucleus caudatus	Cd	Caudate nucleus	Cd	Caudate putamen	CP
Putamen	Pu	Putamen	Put	Putamen	Pu	Caudate putamen	CP
extended pallidum	Pal	Globus pallidus	GP	Globus pallidus	GP	Pallidum	PAL
lateral globus pallidus	LGP					Globus pallidus, external segment	GPe
medial globus pallidus	MGP					Globus pallidus, external segment	GPi
brainstem	Bs			Brainstem		Brain Stem	BS
Interbrain	IBr			Interbrain	IBr	Interbrain	IB
Thalamus	Th			Thalamus	Th	Thalamus	TH
Hypothalamus	Hy			Hypothalamus	Hy	Hypothalamus	HY
Subthalamic nucleus	STh	Nucleus subthalamicus	St	Subthalamic nucleus	STh	Subthalamic nucleus	STN
Midbrain	MBr			Midbrain	MBr	Midbrain	MB
Pars Reticulata	SNR	Substantia nigra, pars reticulata	SNr	Pars Reticulata	SNR	Substantia nigra, reticular part	SNr
Pars Compacta	SNC	Substantia nigra, pars compacta	SNc	Pars Compacta	SNC	Substantia nigra, compact part	SNc
Hindbrain	HBr			Hindbrain	HBr	Hindbrain	HB
Pons	Pons			Pons	Pons	Pons	P
Medulla	MO			Medulla	MO	Medulla	MY
Cerebellum	Cb			Cerebellum	Cb	Cerebellum	CB
Fiber tracts	fiber tracts					Fiber tracts	fiber tracts
Venticular system	VS			cerebral ventricles		Ventricular System	VS
lateral ventricle	LV	Ventriculus lateralis	V1	lateral ventricle	LV	lateral ventricle	VL
third ventricle	3V	Ventriculus quartus	V4	third ventricle	3V	third ventricle	V3
fourth ventricle	4V	Ventriculus tertius	V3	fourth ventricle	4V	fourth ventricle	V4

Nomenclature and abbreviations used in eLemur for mouse lemur brain regions. The eLemur nomenclature is optimized for compatibility with existing English-based frameworks and is shown in comparison to the nomenclature from the Bon’s atlas, NeuroNames, and ARA.

Table S3. Correspondence between Brodmann areas and functional regions

Brodmann area	Names or localization	Relevant NeuroNames IDs
Area 1-3	Primary somatosensory cortex	1006, 1022, 1033, 2391-2393
Area 4	Primary motor cortex	1014, 2394
Area 5	*Subdivision of the parietal region*	1015, 2406
Area 6	Premotor cortex	1020, 2395
Area 7	*Subdivision of the parietal region*	1035, 2407
Area 8	*Subdivision of the frontal region*	1034, 2396
Area 13-16	Insular cortex	1008, 1009, 1010, 2337
Area 17	Primary visual cortex	1026, 2102
Area 18	Secondary visual cortex	1027, 2109
Area 20	Inferior temporal cortex	1025, 2112
Area 21	Middle temporal cortex	1016, 2413
Area 22	Superior temporal cortex	1017, 2101
Area 23	Posterior cingulate cortex	1018, 2107
Area 24	Anterior cingulate cortex	1007, 2113
Area 25	*Subdivision of cingulate region*	1029, 2103
Area 26	*Subdivision of retrosplenial area*	1031, 2114
Area 27	Presubiculum	167, 1039
Area 28	Entorhinal area	168, 1030
Area 29	*Subdivision of retrosplenial area*	2104
Area 30	*Subdivision of retrosplenial area*	2424

The correspondence between Brodmann areas, associated functional brain regions, and NeuroNames ID.

Table S4. Mouse lemur brain ontology

Root	Level 1	Level 2	Level 3	Level 4
grey	Cerebrum	Cerebral cortex	**Neocortex**	Area 1-3
				Area 4
				Area 5
				Area 6
				Area 7
				Area 8
				Area 13-16
				Area 17 (V1)
				Area 18 (V2)
				Area 20
				Area 21
				Area 22
				Area 23
				Area 24
				Area 25
				Area 26-29
				Area 27
				Area 28
				Area 30
			**Hippocampal formation**	Dentate gyrus
				CA fields
				entorhinal area
				Subiculum
				Presubiculum
		Cerebral nuclei	**Striatum**	Caudate nucleus
				Putamen
			**Extended Pallidum**	Lateral Globus pallidus
				Medial Globus pallidus
		**Remaining Region**		
	**Cerebellum**			
	Brainstem	Interbrain	**Thalamus**	
			**Hypothalamus**	Subthalamic nucleus
		**Midbrain**		Pars Compacta
				Pars Reticulata
		Hindbrain	**Pons**	
			**Medulla**	
**Fiber tracts**				
**Ventricular system**				lateral ventricle
				third ventricle
				fourth ventricle

Hierarchical ontology of the mouse lemur brain structures of eLemur. In total, 54 major and sub-divisions are defined and organized hierarchically according to vertebrate embryogenesis. Specifically, levels 1 and 2 illustrate the early stages of brain development, during which the neural tube develops into three vesicles–the forebrain (prosencephalon), midbrain (mesencephalon), and hindbrain (rhombencephalon)–and later into five vesicles that include the cerebrum (telencephalon) and interbrain (diencephalon) originating from the forebrain. The cerebrum is further segmented, following the Allen Mouse Brain Atlas, into two subdivisions: the cerebral cortex (including the neocortex and hippocampus) and the cerebral nuclei (which includes the striatum and pallidum). Levels 3 and 4 include further delineations of the major divisions. The overall ontology is based primarily on three established brain ontology and nomenclature systems: the Allen Mouse Brain Atlas, Brain Maps 4.0, and BrainInfo.

Table S5. Cell counts, densities, and volumes in the mouse lemur and mouse brain regions

	Mouse Lemur							Mouse				
								Erö et al. (2018)				Wang et al. (2020)
	Cell count (DAPI)	Neuron count	PV neuron count	Cell density (number per 100 µm-3)	Neuron density (number per 100 µm-3)	PV neuron density (number per 100 µm-3)	Volume (mm-3)	Cell count (Nissl)	Neuron count	Cell density (number per 100 µm-3)	Neuron density (number per 100 µm-3)	Volume (mm-3)
**Root**	130472181.580	82641372.000	5163155.112	169.8598	115.9231	7.2425	1576.4585	111080000	71760000	232.1202	149.9545	435.2879
**Basic cell groups and regions**	116595697.610	79063250.648	4940518.952	169.8231	124.0763	7.7533	1420.7857	104269365	71760000	243.0774	167.2901	197.4624
**Cerebrum**	83435375.930	62430746.960	3488949.456	173.7428	140.0732	7.8280	938.8211	42954307	25341736	160.2866	94.5642	120.8835
**Cerebral cortex**	63008691.570	49330349.920	2924893.736	183.6444	154.9142	9.1852	684.1721	36851649	22153353	170.2082	102.3206	97.4038
**Isocortex**	57612484.220	45502003.344	2735216.824	188.1825	160.1375	9.6262	608.3288	19845690	10273660	161.1150	83.4056	53.9397
**Area 1-3**	3675139.340	2902007.544	176099.536	172.5419	146.7977	8.9080	39.5375					
**Area 1-3, Layer1**	353755.590	204180.696	8123.312	120.0988	74.6878	2.9714	5.4676					
**Area 1-3, Layer2/3**	1188650.130	983511.640	60684.384	176.4030	157.2647	9.7035	12.5077					
**Area 1-3, Layer4**	386040.850	318317.144	24545.152	197.8415	175.7697	13.5534	3.6220					
**Area 1-3, Layer5**	912315.500	745462.064	54007.536	187.1921	164.8040	11.9398	9.0466					
**Area 1-3, Layer6**	834377.270	650536.000	28739.152	174.1468	146.2932	6.4629	8.8936					
**Area 4**	5399485.700	4061767.840	239727.176	179.7525	145.6926	8.5988	55.7581					
**Area 4, Layer1**	441423.340	194329.456	8744.024	121.5599	57.6599	2.5945	6.7405					
**Area 4, Layer2/3**	1703212.680	1316712.824	77464.112	191.5798	159.5775	9.3882	16.5025					
**Area 4, Layer4**	617862.580	489747.360	34312.512	208.7796	178.3069	12.4925	5.4933					
**Area 4, Layer5**	1266261.390	975748.080	74082.816	186.6545	154.9718	11.7661	12.5926					
**Area 4, Layer6**	1370725.710	1085230.120	45123.712	176.3359	150.4222	6.2545	14.4291					
**Area 6**	2955661.750	2289914.680	112171.792	165.4895	138.1449	6.7670	61.3581					
**Area 6, Layer1**	362441.920	184426.024	7161.488	114.0605	62.5344	2.4283	10.7528					
**Area 6, Layer2/3**	872937.240	712687.352	34163.392	170.3183	149.8225	7.1819	16.7918					
**Area 6, Layer4**	379890.700	308469.632	17044.416	190.0198	166.2465	9.1859	7.4649					
**Area 6, Layer5**	672243.400	536694.064	35197.912	175.5895	151.0423	9.9058	13.5701					
**Area 6, Layer6**	668148.490	547637.608	18604.584	179.1550	158.2155	5.3750	12.7785					
**Area 8**	535800.030	436642.000	21902.000	164.6684	144.5882	7.2526	13.9753					
**Area 8, Layer1**	69151.560	36875.512	1427.824	114.0798	65.5457	2.5379	2.3208					
**Area 8, Layer2/3**	163012.710	143013.536	6415.888	168.3251	159.1125	7.1381	3.9301					
**Area 8, Layer4**	77247.960	65735.824	3798.832	190.9011	175.0344	10.1151	1.8237					
**Area 8, Layer5**	120605.220	101289.760	6809.192	175.5126	158.8209	10.6767	2.9516					
**Area 8, Layer6**	105782.580	89727.368	3450.264	180.0880	164.5869	6.3288	2.9491					
**Area 24**	869496.270	636954.896	41518.736	168.4327	132.9435	8.6657	12.8084					
**Area 24, Layer1**	119049.950	56069.120	2721.440	119.8915	60.8391	2.9530	2.5517					
**Area 24, Layer2/3**	173416.930	141514.880	8442.056	175.4683	154.2798	9.2035	2.5334					
**Area 24, Layer4**	156419.680	126181.616	10561.424	176.5503	153.4522	12.8440	2.2186					
**Area 24, Layer5**	254057.420	197101.224	15372.408	180.9731	151.2764	11.7984	3.4278					
**Area 24, Layer6**	166552.290	116088.056	4421.408	186.8921	140.3550	5.3457	2.0770					
**Area 25**	89797.380	56113.856	2262.896	163.7652	110.2625	4.4465	1.0178					
**Area 25, Layer1**	14819.180	7336.704	341.112	120.7899	64.4329	2.9957	0.2277					
**Area 25, Layer2/3**	12988.840	10852.208	408.216	151.5629	136.4396	5.1323	0.1591					
**Area 25, Layer4**	15298.390	12086.176	430.584	150.4993	128.1084	4.5640	0.1887					
**Area 25, Layer5**	29205.860	18781.664	805.248	183.9695	127.4705	5.4652	0.2947					
**Area 25, Layer6**	17485.110	7057.104	277.736	219.8264	95.5956	3.7622	0.1476					
**Area 5**	6027444.560	4740027.112	332341.880	187.4855	158.8602	11.1383	59.6755					
**Area 5, Layer1**	418038.930	220416.136	13020.040	109.4151	62.1590	3.6717	7.0920					
**Area 5, Layer2/3**	1995236.680	1548624.248	117678.048	184.6746	154.4395	11.7357	20.0548					
**Area 5, Layer4**	696541.250	566488.240	49578.672	217.3492	190.4592	16.6689	5.9487					
**Area 5, Layer5**	1426948.980	1201169.056	95002.488	213.5423	193.6776	15.3183	12.4038					
**Area 5, Layer6**	1490678.720	1203329.432	57062.632	195.1883	169.7673	8.0505	14.1762					
**Area 7**	1298892.650	1025623.128	69831.032	189.4166	161.1508	10.9722	12.7287					
**Area 7, Layer1**	66066.970	34866.120	1496.792	104.6743	59.5195	2.5552	1.1716					
**Area 7, Layer2/3**	406821.610	316147.448	23040.904	184.4454	154.4377	11.2555	4.0942					
**Area 7, Layer4**	151067.060	124023.104	10514.824	219.4045	194.0788	16.4542	1.2781					
**Area 7, Layer5**	353188.150	296338.720	23536.728	214.2437	193.6824	15.3832	3.0600					
**Area 7, Layer6**	321748.860	254247.736	11241.784	191.1248	162.7260	7.1951	3.1249					
**Area 23**	1126240.380	858097.992	66395.680	182.5217	149.8373	11.5937	11.4537					
**Area 23, Layer1**	142359.970	79950.688	4456.824	147.6817	89.3636	4.9815	1.7893					
**Area 23, Layer2/3**	377435.830	304324.096	23739.904	184.5829	160.3557	12.5091	3.7956					
**Area 23, Layer4**	162952.160	127747.376	12842.960	191.1846	161.4896	16.2352	1.5821					
**Area 23, Layer5**	213831.460	168447.816	15489.840	190.0677	161.3251	14.8349	2.0883					
**Area 23, Layer6**	229660.960	177628.016	9866.152	193.9175	161.5999	8.9759	2.1984					
**Area 20**	352900.970	264138.120	13825.288	152.5483	123.0227	6.4391	4.2941					
**Area 20, Layer1**	55337.510	44853.432	1850.952	129.2071	112.8398	4.6565	0.7950					
**Area 20, Layer2/3**	116349.420	99550.648	5062.624	150.6141	138.8498	7.0612	1.4339					
**Area 20, Layer4**	48379.450	38847.624	2315.088	159.2916	137.8149	8.2130	0.5638					
**Area 20, Layer5**	87768.090	57532.360	3247.088	166.6188	117.6791	6.6417	0.9778					
**Area 20, Layer6**	45066.500	23354.056	1349.536	159.7473	89.1953	5.1542	0.5237					
**Area 21**	5535467.160	4474010.080	234754.024	173.7354	151.2972	7.9387	59.1420					
**Area 21, Layer1**	639129.470	430940.024	14759.152	122.6588	89.1100	3.0519	9.6721					
**Area 21, Layer2/3**	1573986.870	1328996.584	69607.352	168.5632	153.3505	8.0319	17.3328					
**Area 21, Layer4**	769837.890	664959.632	40521.496	193.0383	179.6550	10.9479	7.4026					
**Area 21, Layer5**	1452572.010	1232540.176	74541.360	196.5313	179.6780	10.8665	13.7194					
**Area 21, Layer6**	1099940.920	816573.664	35324.664	185.3584	148.2648	6.4139	11.0151					
**Area 22**	7547043.690	5973413.544	326008.008	180.9397	154.3048	8.4214	77.4236					
**Area 22, Layer1**	629830.720	395591.128	14507.512	117.1516	79.2813	2.9075	9.9794					
**Area 22, Layer2/3**	2332159.370	1894362.696	106283.416	178.0843	155.8584	8.7445	24.3088					
**Area 22, Layer4**	1005927.530	842130.968	54212.576	206.8698	186.5992	12.0124	9.0261					
**Area 22, Layer5**	1986081.520	1665664.808	105968.400	203.3726	183.7735	11.6915	18.1274					
**Area 22, Layer6**	1593044.550	1175663.944	45036.104	185.0243	147.1241	5.6359	15.9819					
**Area 27**	308625.080	220034.016	13256.768	219.8862	168.9104	10.1766	2.6053					
**Area 27, Layer1**	49081.830	34090.696	1815.536	197.2552	147.6194	7.8616	0.4619					
**Area 27, Layer2/3**	86327.000	71599.968	4546.296	227.6442	203.4336	12.9172	0.7039					
**Area 27, Layer4**	52360.180	42176.728	2891.064	231.8710	201.2417	13.7944	0.4192					
**Area 27, Layer5**	56382.430	36562.360	2259.168	225.4413	157.5157	9.7328	0.4642					
**Area 27, Layer6**	64473.640	35604.264	1744.704	215.1916	128.0398	6.2743	0.5561					
**Area 28**	250083.610	189026.376	8572.536	154.3280	125.6845	5.6999	3.0080					
**Area 28, Layer1**	49190.820	30081.232	1588.128	111.0049	73.1398	3.8614	0.8226					
**Area 28, Layer2/3**	63454.670	49291.616	2220.024	154.0143	128.9051	5.8057	0.7648					
**Area 28, Layer4**	47858.720	39168.232	1946.016	167.8438	148.0055	7.3534	0.5293					
**Area 28, Layer5**	46417.630	40040.584	1679.464	185.2588	172.1853	7.2221	0.4651					
**Area 28, Layer6**	43161.770	30444.712	1138.904	187.9639	142.8522	5.3439	0.4262					
**Area 17**	15017524.500	12141685.920	755322.624	219.4395	191.1592	11.8918	127.3157					
**Area 17, Layer1**	1280833.180	843318.336	43982.944	150.2835	106.6130	5.5604	15.9323					
**Area 17, Layer2/3**	5278219.620	4353666.512	271743.240	213.3869	189.6423	11.8369	46.0859					
**Area 17, Layer4**	3456372.190	2979939.520	199953.144	249.3462	231.6272	15.5421	25.7305					
**Area 17, Layer5**	2378431.680	2010174.880	129380.240	242.2696	220.6185	14.1996	18.2231					
**Area 17, Layer6**	2623667.830	1954586.672	110263.056	228.1732	183.1515	10.3320	21.3439					
**Area 18**	3012058.020	2383342.088	142344.360	200.8737	171.2561	10.2282	27.8337					
**Area 18, Layer1**	229571.000	151530.152	7247.232	130.2851	92.6567	4.4315	3.2708					
**Area 18, Layer2/3**	964234.530	771530.104	47904.800	194.5911	167.7618	10.4164	9.1979					
**Area 18, Layer4**	379247.140	326282.016	22638.280	229.7770	212.9988	14.7784	3.0637					
**Area 18, Layer5**	840119.140	717822.672	45567.344	225.0013	207.1386	13.1491	6.9308					
**Area 18, Layer6**	598886.210	416177.144	18986.704	206.9982	154.9888	7.0709	5.3704					
**Area 13-16**	1152263.040	951996.992	45496.512	139.1343	123.8563	5.9192	15.3726					
**Area 13-16, Layer1**	183973.390	116034.000	3308.600	109.6288	74.4997	2.1243	3.1150					
**Area 13-16, Layer2/3**	384437.140	338981.448	14373.304	139.9256	132.9376	5.6367	5.0999					
**Area 13-16, Layer4**	147902.890	128748.344	6872.568	147.7263	138.5551	7.3961	1.8584					
**Area 13-16, Layer5**	246251.660	212742.048	12604.368	152.2265	141.6981	8.3952	3.0028					
**Area 13-16, Layer6**	189697.960	155491.152	8337.672	153.3272	135.4135	7.2611	2.2965					
**Area 26-29**	1394385.190	1077591.448	79781.064	205.2070	170.8691	12.6505	12.6131					
**Area 26-29, Layer1**	178229.790	101114.544	5948.024	164.2434	100.3971	5.9058	2.0143					
**Area 26-29, Layer2/3**	445558.040	372757.128	27699.040	209.7821	189.0993	14.0517	3.9424					
**Area 26-29, Layer4**	233489.450	191669.528	16041.584	213.0609	188.4470	15.7719	2.0342					
**Area 26-29, Layer5**	250734.090	202473.272	16539.272	215.5276	187.5240	15.3181	2.1594					
**Area 26-29, Layer6**	286373.820	209576.976	13553.144	215.8510	170.2018	11.0068	2.4627					
**Area 30**	453678.660	357837.672	25016.744	219.4603	186.5063	13.0388	3.8373					
**Area 30, Layer1**	62911.450	46324.128	3284.368	186.3068	147.8108	10.4797	0.6268					
**Area 30, Layer2/3**	143742.240	120011.776	9778.544	224.5957	202.0415	16.4623	1.1880					
**Area 30, Layer4**	52059.160	44611.112	3534.144	234.6724	216.6745	17.1652	0.4118					
**Area 30, Layer5**	122143.190	101871.328	6766.320	227.7218	204.6384	13.5921	0.9956					
**Area 30, Layer6**	72822.620	45019.328	1653.368	219.7699	146.3864	5.3761	0.6151					
**Remaining region**	5396207.350	3828346.576	189676.912	146.0431	111.6358	5.5310	75.8433					
**Hippocampal formation**	8836869.410	4942541.392	234129.584	127.3087	76.7202	3.6343	128.8459	6857094	4446636	167.0549	108.3305	19.1897
**Regio Entorhinalis**	3110036.570	2191689.336	117029.376	124.6451	94.6430	5.0536	46.3149	1708551	1082511	143.8010	91.1100	5.1126
**Ammon's horn**	2734036.640	1193064.384	56920.968	120.1379	56.4858	2.6949	42.2430	2361489	1564060	162.8190	107.8382	7.6370
**Dentate Gyrus**	1547066.340	757671.264	26576.912	136.7816	72.1771	2.5318	20.9948	1445049	1032255	233.1788	166.5687	3.0101
**Subiculum**	771702.830	363811.792	15827.224	121.6931	61.8148	2.6892	11.7710	809340	440320	153.8793	83.7177	1.0674
**Presubiculum**	645713.850	427185.928	17046.280	167.8818	119.6686	4.7752	7.1395	211652	127654	170.3008	102.7138	0.3161
**Indusium Griseum**	28313.180	9118.688	728.824	137.3130	47.6492	3.8084	0.3827	6234	3643	166.3786	97.2277	0.0663
**Cerebral nuclei**	11589814.950	8157855.648	329926.136	171.1712	129.8166	5.2501	125.8030	6102658	3188383	118.5553	61.9402	23.7599
**Striatum**	9279296.150	7173001.928	235973.080	175.2831	145.9909	4.8027	98.3867	5062235	2695250	121.0052	64.4259	39.6438
**Caudate Nucleus**	3562877.910	2837940.000	82839.888	185.8566	159.5071	4.6560	35.7041					
**Putamen**	2718259.040	2137793.640	59579.032	170.9339	144.8448	4.0367	29.5184					
**Caudoputamen**								2889135	1470191	117.6587	59.8729	11.4371
**Pallidum**	2310518.800	984853.720	93953.056	156.4336	71.8442	6.8538	27.4164	1040423	493133	107.9240	51.1531	4.3221
**Globus Pallidus, external segment**	609216.040	179327.984	30297.456	150.1802	47.6309	8.0472	7.5299	185535	85521	104.6917	48.2569	0.7383
**Globus Pallidus, internal segment**	212961.270	64009.760	8652.688	148.0214	47.9369	6.4800	2.6706	33786	15193	102.8493	46.2496	0.2034
**Brain stem**	24204295.060	11597858.328	855262.848	154.8238	79.9324	5.8945	309.1146	14694541	6811201	129.6194	60.0811	52.8044
**Interbrain**	9207476.930	4313441.392	236295.552	161.8254	81.6827	4.4747	105.6146	5399178	2890478	150.6396	80.6457	15.9320
**Thalamus**	6548120.930	2869980.296	173819.864	156.5825	73.9444	4.4784	77.6254	3036911	1568511	152.2048	78.6111	18.8635
**Hypothalamus**	2659356.000	1443461.096	62475.688	176.3663	103.1440	4.4643	27.9892	2362267	1321967	148.6741	83.2007	13.8481
**Subthalamic Nucleus**	41500.970	20200.168	1504.248	181.1562	95.0057	7.0748	0.4252	36110	19161	176.3210	93.5610	0.0978
**Midbrain**	8413919.010	4215115.392	318182.936	163.3158	88.1534	6.6544	95.6314	4725452	1998739	133.7073	56.5546	33.1406
**Substantia nigra, compact part**	125374.830	64608.104	6263.040	140.7422	78.1449	7.5753	1.6535	60832	30838	136.5908	69.2430	0.1008
**Substantia nigra, reticular part**	321328.470	138305.072	17553.288	123.9129	57.4652	7.2933	4.8135	131191	61568	98.1670	46.0698	0.6836
**Hindbrain**	6582899.120	3069301.544	300784.360	137.3798	69.0153	6.7633	107.8686	4569911	1921984	108.3343	45.5626	20.9193
**Pons**	2274894.640	1054425.656	126172.296	137.0359	68.4366	8.1891	30.8147	1681695	715713	103.7634	44.1607	14.6422
**Medulla**	4308004.480	2014875.888	174612.064	137.5622	69.3220	6.0075	77.0539	2888216	1206271	111.1862	46.4372	27.6195
**Cerebellum**	8956026.620	5034645.360	596306.648	179.0722	108.4630	12.8464	172.8501	46620517	39607063	979.3378	832.0091	47.1311
**fiber tracts**	12905872.660	3292806.328	200035.160	173.3175	47.6454	2.8944	142.3052	6810635	0	137.3392	0.0000	47.0055
**ventricular systems**	970611.310	285315.024	22601.000	147.6693	43.4080	3.4385	13.3676					2.7920
**lateral ventricle**	330338.310	110660.088	5871.600	195.5094	65.4937	3.4751	3.6011					1.7370
**third ventricle**	481325.790	117348.120	8689.968	138.1612	33.6840	2.4944	6.9676					0.9700
**fourth ventricle**	158947.210	57306.816	8039.432	113.5786	40.9496	5.7447	2.7989					1.2766

Count and densities of total cells, neurons, and PV-neurons, along with the volumes of each brain region in the mouse lemur. Corresponding data for mouse brain regions, extracted from previously published sources, are also included.

Movie S1. Serial block face images with annotations
Serial block face images of the mouse lemur brain with overlaid annotations on the left hemisphere tissue.

Movie S2. Serial brain images immunostained with multiplex markers and annotations
Aligned images of the mouse lemur brain immunostained with the markers such as SMI-99, VGLUT2, NeuN and DAPI. Separate fluorescent channel images of each marker are followed by the merged channels with overlaid annotations.

Movie S3. Exploring open source data on eLemur website
The interactive platform of eLemur aims to provide users with the IHC image datasets, 2D reference atlas, 3D interactive atlas, and cell atlas

SI References

1. Palazzi, X. & Bordier, N. The Marmoset Brain in Stereotaxic Coordinates. (2008) doi:10.1007/978-0-387-78385-7.
